# Self‐Reported Motor and Non‐Motor Symptoms in People With Functional Gait Disorder: A Cross‐Sectional Study

**DOI:** 10.1002/brb3.70208

**Published:** 2025-02-06

**Authors:** Sara Issak, Gavin Williams, Richard A. Kanaan, Natalie A. Fini, Glenn Nielsen

**Affiliations:** ^1^ Department of Physiotherapy Epworth Healthcare Melbourne Australia; ^2^ Department of Physiotherapy, Melbourne School of Health Sciences The University of Melbourne Victoria Australia; ^3^ Department of Psychiatry University of Melbourne, Austin Health Victoria Australia; ^4^ Neurosciences Research Centre, Molecular & Clinical Sciences Research Institute St George's, University of London London UK

**Keywords:** functional gait disorder, functional neurological disorders, motor symptoms, nonmotor symptoms, self‐report, survey

## Abstract

**Background:**

Functional gait disorder is a common presentation of functional neurological disorder. Altered gait is the defining feature, along with a range of associated motor and nonmotor symptoms. The aim of this study was to explore the prevalence and impact of these symptoms in people with functional gait disorder.

**Methods:**

A total of 156 people with functional gait disorder completed an online survey that included demographic information, self‐reported symptoms, and standardized questionnaires.

**Results:**

Weakness (85.9%) and reduced balance (80.1%) were the most prevalent motor symptoms, while fatigue (85.9%), somatosensory (69.9%), and cognitive (69.9%) symptoms were the most prevalent nonmotor symptoms. Logistic regression indicated that dependent ambulation had the greatest association with fear of falling and functional seizures (*X* ^2^ (11, *n* = 128) = 40.68, *p* < 0.001). Stepwise regression indicated that functional seizures, muscle rigidity, depression, fear of falling, pain, and speech symptoms were associated with reduced participation in work and social function (adjusted *R* ^2^ = 0.39, *F* (6, 120) = 14.31, *p* < 0.001). Stepwise regression revealed that lower physical quality of life was associated with pain, bradykinesia, fatigue, and dystonia (adjusted *R* ^2^ = 0.32, *F* (4, 122) = 15.92, *p* < 0.001) while depression, anxiety, and functional seizures were associated with reduced mental quality of life (adjusted *R* ^2^ = 0.46, *F* (3, 123) = 36.89, *p* < 0.001).

**Conclusions:**

Motor and nonmotor symptoms are highly prevalent in people with functional gait disorder and are associated with high levels of disability, reduced participation in work and social function, and reduced quality of life.

## 1 Background

Functional neurological disorders (FNDs) are characterized by sensory, motor, and cognitive symptoms from alterations in brain network functioning, rather than structural changes (Hallett et al. 2022). Motor presentations of FND (motor‐FND) include tremor, weakness, dystonia, and altered gait, commonly known as functional gait disorder (FGD) (Edwards et al. 2012). In outpatient settings, FGD represents up to 37% of motor‐FND presentations (Baik et al. 2007). Examples of FGD phenotypes include ataxia with uncoordinated movements, abnormal posturing of the trunk and/or limbs, scissoring gait, or dragging of lower limbs (Fung 2016).

In addition to the gait impairment, people with FGD may have other concurrent motor and nonmotor symptoms as part of the wide spectrum of FND, such as weakness, tremor, dystonia, pain, anxiety, and fatigue (Gelauff et al. 2018; Tinazzi et al. 2020, 2021; Věchetová et al. 2018). Motor symptoms, such as weakness or tremor may impact movement, while nonmotor symptoms encompass a whole range of factors that can influence psychological, sensory, cognitive, and communication domains (Issak et al. 2023). An international online survey of 1048 respondents found that co‐existing symptoms are common in FND, including high prevalence rates of fatigue (93%), memory difficulties (80%), headache (70%), depression (43%), and anxiety (51%) (Butler et al. 2021).

Previous studies found that nonmotor symptoms have a greater impact on disability and quality of life than motor symptoms. Gelauff et al. (2018) investigated 181 participants with motor‐FND and found that quality of life was negatively associated with fatigue and depression, but not with motor symptom severity. In 61 people with motor‐FND, Věchetová et al. (2018) found that health‐related quality of life negatively correlated with depression, anxiety, and pain, while no correlation was found between motor symptom severity and health‐related quality of life.

Other co‐existing symptoms that may impact ambulation and disability include sensory impairment, cognitive difficulties, and functional seizures (Baker et al. 2021; Goldstein et al. 2020; Revell 2019; Teodoro, Edwards, and Isaacs 2018), but their relative prevalence and impact in FGD remain unknown. Additionally, there may be factors that specifically interact with gait, such as fear of falling (Scheffer et al. 2008) and kinesiophobia (fear of movement) (Vlaeyen et al. 1995), yet there is no empirical data on the effect of these symptoms in people with FGD.

Co‐existing symptoms are common and associated with large burden, but it is unclear which of these symptoms exert important influence in people with FGD. Therefore, the aim of this study was to explore the prevalence and severity of self‐reported motor and nonmotor symptoms and the impact on ambulation, participation in work and social function, and quality of life (QOL).

## 2 Methods

### 2.1 Design and Ethics

An observational cross‐sectional study was conducted through an online survey of people living with FGD. The survey was advertised through FND‐peer support groups, social media, and within professional FND networks. The survey was piloted by two volunteers with FGD and six clinicians working in the field. Their feedback was incorporated into the final survey. Data were collected over a 7‐month period (February 1 to September 1, 2022) using the secure, web‐based software platform, Research Electronic Data Capture tool (REDCap) hosted at The University of Melbourne. Human research ethics approval was obtained from the University of Melbourne's Office of Research Ethics and Integrity (Reference Number: 2021‐22778‐23936‐3).

### 2.2 Participants and Recruitment

Adults (≥ 18 years) with a formal diagnosis of FND from a medical professional, and impaired or altered walking, were eligible for participation in the study. People were excluded if they were nonambulant. Participants were recruited via self‐selection sampling. Potential participants completed screening questions prior to commencing the survey to ensure eligibility and provide informed consent (See File S1 for a copy of the survey questions). An online voucher valued at 30AUD was provided to respondents who completed the survey.

### 2.3 Survey Content

The survey was provided in English and made up of three sections: consent, demographics, and survey questions. We compiled a comprehensive list of eight motor and 16 nonmotor symptoms commonly reported in people with FND (Butler et al. 2021), and also provided an option for respondents to include “other” symptoms. Respondents were asked to endorse all of the symptoms that they regularly experience at the time of the survey and the rate of occurrence on an 8‐point ordinal scale (symptoms occur constantly every hour, most of the day, most days of the week, once a week, once a month, once every 3 months, once in 12 months, or never). The symptoms were also rated in terms of perceived severity on a sliding scale between 0 and 100, with 100 indicating the highest severity.

A battery of standardized outcome measures were included in the survey that were informed by recommendations from a systematic review of outcome measurement in FND (Pick et al. 2020). These were the 36‐Item Short Form Survey (SF36) (RAND Health Care 2021), Patient Health Questionnaire (PHQ15) (Kroenke, Spitzer, and Williams 2002), Hospital Anxiety and Depression Scale (HADS) (RehabMeasures Database 2021), and Work and Social Adjustment Scale (WSAS) (Mundt et al. 2002). In the absence of outcome measure recommendations for a particular domain, measures were selected on the basis of face validity, use in other FND studies, and reliability in other neurological populations. These were the Falls Efficacy Scale (FES) (RehabMeasures Database 2021), Tampa Scale for Kinesiophobia (TSK) (Miller et al., 1991), Functional Ambulation Category (RehabMeasures Database 2021), and Functional Mobility Scale (RehabMeasures Database 2021). The Checklist for Reporting Of Survey Studies was used to report findings (Sharma et al. 2021).

### 2.4 Statistical Analyses

A power calculation was conducted using G*Power (version 3.1.9.7) to determine the minimum sample size required to detect the association between self‐reported symptoms and measures of ambulation, participation, and QOL. The required sample size to achieve 90% power for detecting a moderate effect (0.3), at a significance of 0.05, was 109 participants. An additional 20% inflation factor was added to account for dropouts, resulting in a final target sample size of *n* = 131. Data analysis was completed using all of the available responses, including partial survey responses.

Data were analyzed utilizing IBM SPSS Version 28.0.1.0. Descriptive statistics were used to summarize the data. Categorical variables were reported as counts (percentages), and continuous variables were reported as means (standard deviations). All data were tested for normality using the Kolmogorov–Smirnov statistic.

Symptom occurrence data (reported by participants on an 8‐point ordinal scale) for each symptom type were collapsed into two groups: “constant” (categories: the symptom is experienced constantly every hour, most of the day, or most days of the week) or “episodic/never” (categories: the symptom is experienced once a week, once a month, once every 3 months, once in 12 months, or never). All statistical analyses were completed using this recoding to ensure that symptom impact was explored in those experiencing the symptom regularly.

The Functional Ambulation Category scores were dichotomized into dependent ambulation (scores 0 to 3 reflecting the need for supervision or assistance by another person to walk) and independent ambulation (score of 4 or 5). Component summaries from the SF36 were used as they reduce the number of scores derived from eight domains to two, with the advantage of having smaller confidence intervals than the individual health domain scales, limiting floor and ceiling effects (Hooker, S.A. 2013). The physical component summary combines items from the physical functioning, role‐physical, bodily pain, and general health scales. The Mental Health Component Summary combines items from the energy, social functioning, role‐emotional, and wellbeing scales (Granger and Johnson 2013). Physical and mental summary scores were derived using an orthogonal‐factor analytic model from the eight domains of the SF36 scale (Laucis, Hays, and Bhattacharyya 2015; Ware, Kosinski, and Keller 2023)

The aim of the analyses was exploratory, to examine if any of the (constant) motor and nonmotor symptoms had a significant impact on independence in ambulation, participation in work and social function, and quality of life. We hypothesized that nonmotor symptoms may have a greater impact on these three outcome domains than motor symptoms.

Binary logistic regression analysis was used to determine which self‐reported symptoms had the greatest impact on dependent ambulation. In step one, a chi‐squared test for independence (with Yates continuity correction) was used to explore which constantly experienced symptoms were associated with dependent ambulation. In step two, symptoms that were statistically associated with dependent ambulation (*p* < 0.05) in step one were entered into a binary logistic regression model.

A stepwise, multivariable, linear regression model was used to identify which constant symptoms were associated with work and social function (WSAS), physical quality of life (SF36 Physical Summary score), and mental quality of life (SF36 Mental Summary score). Both forward and backward stepwise regressions were performed as a check to ensure that the resulting set of predictors was the same and to account for possible collinearity between predictor variables. Symptoms for the multivariable model were selected based on significance in univariate analyses. The univariate analyses dichotomized participants into constant versus episodic (as defined above) groups for each symptom type. Mean WSAS and SF36 Summary scores for the two groups were compared using independent sample *t*‐tests. Where a significant difference was found (*p* < 0.05), the symptom was selected for multivariable analysis.

## 3 Results

### 3.1 Demographic Characteristics

**FIGURE 1:**
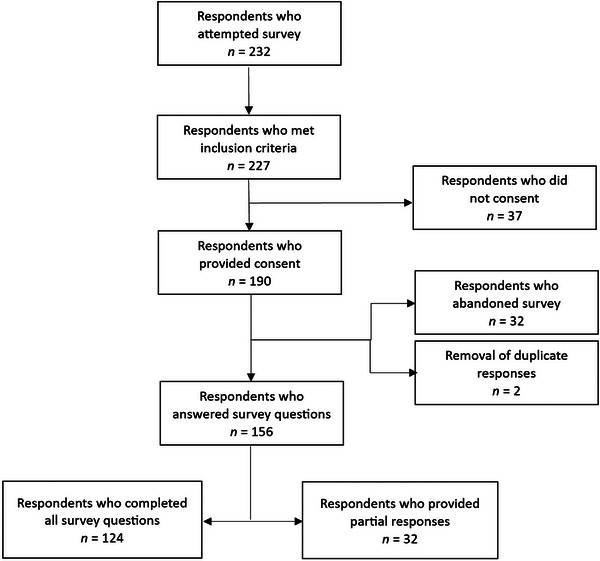
Flow chart of survey responses.

A total of 156 respondents completed the survey (Figure 1). The majority of respondents resided in the United Kingdom (53.8%), Australia (19.8%), and the United States of America (18.6%). The remaining respondents resided in Canada (3.8%), Ireland (1.92%), Sweden (0.6%), Denmark (0.6%), and New Zealand (0.6%). The cohort was predominately female (90.4%) with a mean age of 43.5 years (SD 13.6). The majority (92.9%) were diagnosed by a neurologist and had a mean symptom duration of 5.5 years (SD 7.2). At the time of survey completion, 28.8% of respondents were receiving treatment, while 39.7% of respondents had previously received treatment. Therapy disciplines involved in previous treatment included physiotherapy (34%), psychology (14.7%), and occupational therapy (13.5%). Forty respondents (25.6%) reported a comorbidity that could impact gait, such as sciatica or osteoarthritis. See Table S1, for more details.

Table 1 summarizes the mean scores from the questionnaires used in the survey. The cohort reported severe impact on work and social function (WSAS mean 25.4, SD 10.0); high levels of anxiety (HADS Anxiety mean 11.2, SD 4.6); and poor physical and mental quality of life, as indicated by the SF36 Summary scores. Respondents had high levels of fear of falling as indicated by a mean FES score of 42.1 (SD 14.1) and a high degree of kinesiophobia with a mean TSK score of 42.5 (SD 8.3). More than half of the cohort (54.7%) required a walking aid, such as a walking frame or walking stick, to mobilize 50 m, and nearly half of the respondents (46.9%) required supervision or assistance to walk. See Tables S2–S4 for more detailed data on SF36 domains and mobility scales.

**TABLE 1:** Results from standardized outcome measures and questionnaires.

Measure	Outcome, *n* (%)	Mean score (SD)
Falls efficacy scale (FES)		
Respondents with available data	133 (85.2%)	42.1 (14.1)
Hospital anxiety and depression scale (HADS)		
Respondents with available data	131 (83.9%)	
Anxiety subscale		11.2 (4.6)
Depression subscale		9.2 (4.4)
Tampa scale for kinesiophobia (TSK)		
Respondents with available data	129 (82.7%)	42.5 (8.3)
Work and social adjustment scale (WSAS)		
Respondents with available data	127 (81.4%)	25.4 (10.0)
Functional ambulation category (FAC)		
Respondents with available data	128 (82.1%)	
Respondents who were dependently ambulant	60 (46.9%)	
Respondents who were independently ambulant	68 (53.1%)	
SF36 Physical summary score (PCS‐36)		
Respondents with available data	127 (81.4%)	29.2 (8.2)
SF36 Mental summary score (MCS‐36)		
Respondents with available data	127 (81.4%)	33.9 (12.6)
Patient health questionnaire (PHQ15)		
Respondents with available data	129 (82.7%)	14.5 (6.2)

*Note*: Higher FES scores indicate a greater fear of falling with 16–19 = low concern, 20–27 = moderate concern, and 28–64 = high concern (Delbaere et al. 2010). Higher HADS scores indicate greater severity with 0–7 = normal, 8–10 = borderline, and 11–21 = high (abnormal) (Zigmond and Snaith 1983). A higher TSK value indicates a high degree of kinesiophobia with a score of > 37 considered predictive of poorer health outcomes (Vlaeyen et al. 1995). Higher WSAS scores are associated with greater functional impairment with 0–10 = none to minimal functional impairment, 10–20 = moderate, and > 20 = severe (Mundt et al. 2002). Dependent ambulant is reflected by respondents scoring 0–3 on the scale. Independent ambulation is reflected by those scoring 4–5 on the FAC questionnaire. Higher SF36 scores indicate a more favorable health status (RAND Health Care 2021). Higher PHQ15 scores indicate greater somatic symptom severity with 0–4 = minimal symptom severity, 5–9 = low, 10–14 = medium, and 15–30 = high (Kroenke, Spitzer, and Williams 2002).

### 3.2 Prevalence of Self‐Reported Symptoms

**FIGURE 2:**
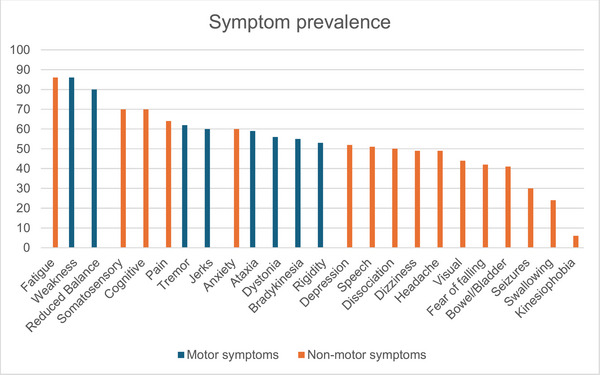
Self‐reported prevalence of motor and nonmotor symptoms (*n* = 156).

Weakness (85.9%), reduced balance (80.1%), and tremor (61.5%) were the most prevalent motor symptoms. The mean severity scores for motor symptoms ranged from 52.8/100 (tremor) to 65.1/100 (bradykinesia). The median number of motor symptoms reported by each participant was 5 out of a maximum of 8 (IQR = 4–7). The most prevalent nonmotor symptoms were fatigue (85.9%), somatosensory symptoms (69.9%), and cognitive symptoms (69.9%). The mean nonmotor symptom severity ranged from 56.8/100 (visual symptoms) to 75.9/100 (fatigue). The median number of nonmotor symptoms reported by each participant was 8 out of a maximum of 16 (IQR = 5–10). Other motor and nonmotor symptoms nominated by respondents included muscle cramping (*n* = 1), tics (*n* = 2), feeling faint with postural change (*n* = 1), and pressure in joints (*n* = 1). See Table 2 and Figure 2 for more details.

**TABLE 2:** Self‐reported prevalence and severity of motor and nonmotor symptoms (*n* = 156).

	Prevalence			
Symptom	Symptoms experienced constantly, *n* (%)	Symptoms experienced episodically, *n* (%)	Total (constant and episodic), *n* (%)	Severity rating from 100, mean (SD)
**Motor symptoms**				
Weakness	117 (75.0)	17 (10.9)	134 (85.9)	64.7 (19.9)
Reduced balance	102 (65.4)	23 (14.7)	125 (80.1)	64.3 (21.1)
Tremor	75 (48.1)	21 (13.5)	96 (61.5)	52.8 (21.5)
Jerks	74 (47.4)	20 (12.8)	94 (60.3)	55.3 (22.7)
Ataxia	73 (46.8)	19 (12.2)	92 (59.0)	62.7 (20.9)
Dystonia	57 (36.5)	30 (19.2)	87 (55.8)	59.0 (21.1)
Bradykinesia	72 (46.2)	14 (8.9)	86 (55.1)	65.1 (22.8)
Rigidity	61 (39.1)	21 (13.5)	82 (52.6)	64.5 (19.3)
Other motor	13 (8.30)	4 (2.6)	17 (10.9)	79.9 (11.3)
**Nonmotor symptoms**				
Fatigue	128 (82.1)	6 (3.8)	134 (85.9)	75.9 (16.9)
Somatosensory	95 (60.9)	14 (8.9)	109 *(69.9)*	67.5 (21.1)
Cognitive	99 (63.5)	10 (6.4)	109 (69.9)	69.1 (18.4)
Pain	97 (62.2)	3 (1.9)	100 (64.1)	70.2 (18.7)
Anxiety	80 (51.3)	12 (7.7)	92 (59.0)	69.6 (19.0)
Depression	59 (37.8)	22 (14.1)	81 (51.9)	NR
Speech	54 (34.6)	26 (16.7)	80 (51.3)	63.6 (19.8)
Dissociation	49 (31.4)	29 (18.6)	78 (50.0)	65.1 (22.1)
Dizziness	52 (33.3)	25 (16.0)	77 (49.4)	63.1 (21.4)
Headache	43 (27.6)	33 (21.2)	76 (48.7)	65.8 (22.1)
Visual	47 (30.1)	21 (13.5)	68 (43.6)	56.8 (22.2)
Fear of falling	51 (32.7)	15 (9.6)	66 (42.3)	64.6 (21.4)
Bowel/bladder	50 (32.1)	14 (8.9)	64 (41.0)	64.8 (23.9)
Functional seizures	20 (12.8)	27 (17.3)	47 (30.1)	63.8 (24.4)
Swallowing	24 (15.4)	13 (8.3)	37 (23.7)	58.3 (22.9)
Kinesiophobia	7 (4.5)	2 (1.3)	9 (5.8)	69.2 (23.7)
Other nonmotor	14 (9.0)	1 (0.6)	15 (9.6)	69.6 (23.6)

*Note*: Prevalence as a constant symptom indicates the proportion of respondents reporting the symptom constantly; experienced every hour, most of the day, or most days of the week. Episodic prevalence indicates symptoms experienced once a week, once a month, once every 3 months, once in 12 months, or never. Severity rating was measured on a sliding scale from 0 to 100, with 100 indicating highest severity. NR: not recorded—the severity rating of depression was omitted in error from the online survey. See Table S13 for correlation matrix between all symptoms.

### 3.3 Symptoms Associated With Dependent Ambulation

In univariate analysis, the chi‐square test for independence found significant associations between dependent ambulation (needing supervision/assistance to walk) and the following constant motor symptoms: tremor, bradykinesia, dystonia, weakness, balance impairment, and jerks. Constant nonmotor symptoms significantly associated with dependent ambulation were functional seizures, fear of falling, anxiety, and fatigue. The binary logistic regression model indicated that fear of falling (OR 0.36, 95% CI 0.14, 0.94) and functional seizures (OR 0.16, 95% CI 0.03, 0.85) were statistically significant (*X* ^2^ (11, *n* = 128) = 40.68, *p* < 0.001), explaining 36.3% (Nagelkerke *R* ^2^) of the variance in dependent ambulation. See Tables S5 and S6 for full details.

### 3.4 Symptoms Associated With Impaired Work and Social Function

All constant motor symptoms were associated with significantly higher (worse) mean WSAS scores; and all, apart from two, constant nonmotor symptoms (headache and sensory symptoms) were associated with worse WSAS scores. The multivariable stepwise linear regression analysis found that the following symptoms were significantly associated with impaired work and social function: functional seizures (*β* = − 0.23, *p* = 0.002), rigidity (*β* = − 0.19, *p* = 0.017), depression (*β* = − 0.19, *p* = 0.012), fear of falling (*β* = − 0.17, *p* = 0.019), pain (*β* = − 0.19, *p* = 0.014), and speech symptoms (*β* = − 0.16, *p* = 0.037). The model explained 39% of the variance in WSAS scores (adjusted *R* ^2^ = 0.39, *F*(6, 120) = 14.31, *p* < 0.001). See Tables S7 and S8 for full details.

### 3.5 Symptoms Associated With Impaired Physical Quality of Life

Constant symptoms significantly associated with lower mean SF36 Physical Summary scores were bradykinesia, rigidity, weakness, jerks, reduced balance, dystonia, tremor, ataxia, fatigue, pain, functional seizures, bowel and bladder symptoms, visual symptoms, fear of falling, headache, dizziness, swallowing, and speech symptoms. Subsequent multivariable stepwise linear regression analysis found that the following symptoms were associated with impaired physical quality of life: pain (*β* = 0.29, *p* < 0.001), bradykinesia (*β* = 0.25, *p* < 0.001), fatigue (*β* = 0.21, *p* = 0.009), and dystonia (*β* = 0.16, *p* = 0.041) The model explained 32% of the variance in SF36 Physical Summary scores (adjusted *R* ^2^ = 0.32, *F*(4, 122) = 15.92, *p* < 0.001). See Tables S9 and S10 for full details.

### 3.6 Symptoms Associated With Impaired Mental Quality of Life

A lower mean SF36 Mental Summary score was associated with the following constant symptoms: jerks, depression, kinesiophobia, anxiety, functional seizures, fear of falling, dissociation, cognitive, and speech symptoms. The multivariable stepwise linear regression analysis found that the following symptoms were associated with impaired mental quality of life: depression (*β* = 0.42, *p* < 0.001), anxiety (*β* = 0.35, *p* < 0.001), and functional seizures (*β* = 0.16, *p* = 0.016). The model explained 46% of the variance in the SF36 Mental Summary score (adjusted *R* ^2^ = 0.46, *F*(3, 123) = 36.89, *p* < 0.001). See Tables S11 and S12 for further details, and Box 1 for a summary of the statistical findings.

BOX 1 **Motor and non‐motor symptoms associated with ambulation, participation, and quality of life in people with functional gait disorder.**

**Table:** 

Symptoms associated with ambulation, participation, and quality of life in people with functional gait disorder	
Dependent Ambulation (Requiring assistance to walk)	Fear of falling Functional seizures
Impaired Work and Social Participation	Functional seizures Muscle rigidity Depression Fear of falling Speech disturbance
Reduced Mental Quality of Life	Depression Anxiety Functional Seizures
Reduced Physical Quality of Life	Pain Fatigue Bradykinesia Dystonia

Note. Box 1 is a summary of the statistical findings from this study, indicating which motor and non‐motor symptoms were significantly associated with reduced quality of life, participation and independent ambulation, in this cohort with functional gait disorder.

## 4 Discussion

In this study, we explored self‐reported symptoms in people with FGD, which we defined as a self‐reported gait impairment and a diagnosis of FND. The cohort was comparable to previously reported studies, with similar mean age and symptom duration (Tinazzi et al. 2021; Butler et al. 2021). Most participants reported experiencing multiple symptoms, with the median number of symptoms endorsed being 13, from a possible total of 24. Both motor and nonmotor symptoms were common. Fatigue and weakness were the two most common symptoms, each endorsed by 85.9% of the cohort.

Fear of falling and functional seizures were significantly associated with dependent gait (requiring assistance from another person to walk) in a multivariable logistic regression model, explaining 36% of the variance of the Functional Ambulation Category. Fear of falling is not commonly assessed or reported in studies of FND, therefore, this is a novel finding that has important clinical implications. In a systematic review of 28 studies of community‐dwelling elderly people, fear of falling was associated with a decline in physical and mental function, an increased risk of falling, and reduced health‐related quality of life (Scheffer et al. 2008). This is important as fear of falling, in people with FGD, may prove to be a responsive treatment target. One recent meta‐analysis of 31 studies found that combined interventions (physical exercise and cognitive intervention) had positive effects on fear of falling in older adults (Hu et al. 2024). This treatment approach aligns with the recommended interdisciplinary treatment advised for people with FND more broadly, so can be applied in cases of FGD with fear of falling.

The importance of functional seizures in dependent ambulation was an unexpected finding. One explanation may be that the postictal period of a functional seizure is often associated with an exacerbation of other symptoms, such as fatigue and limb weakness (Ettinger et al. 1999), leaving the person dependent on help from others. Another explanation may be related to avoidance of independent walking due to the potential unpredictability of a functional seizure, which often results in sudden loss of motor control and awareness, leaving the person vulnerable to falls, injuries, or loss of dignity in public situations.

In previous studies, motor symptoms have been found to have limited importance in explaining reduced quality of life, when compared to the nonmotor symptoms of fatigue, pain, anxiety, and depression (Gelauff et al. 2018; Věchetová et al. 2018). In our study, we took a broad view of quality of life, taking into account work and social function (WSAS), and from the SF36 we calculated separate physical and mental quality of life summary scores. Using regression analysis, we determined which self‐reported symptoms were significantly associated with these health domains. Work and social function was significantly associated with a mixture of physical (motor and nonmotor) and psychological symptoms. These were muscle rigidity, speech disturbance, functional seizures, pain, fear of falling, and depression. Physical quality of life was associated with only physical symptoms, which were a mixture of motor symptoms (bradykinesia and dystonia) and nonmotor symptoms (pain and fatigue). Mental quality of life was associated with psychological symptoms: depression and anxiety, but also functional seizures, which occupy a middle ground between motor and nonmotor, as well as physical and psychological domains (Espay et al. 2018; Stone and Edwards 2011). In general, nonmotor symptoms were more commonly associated with our chosen domains of quality of life. However, we found that motor symptoms are not unimportant. It is possible that previous studies underestimated the impact of motor symptoms because they were assessed using scales for quality of life that are weighted toward measures of mental health.

Our findings, and those of similar studies, highlight some problems with the binary division into motor and nonmotor symptoms in FND. For example, we found that the motor problem of gait impairment was more closely related to nonmotor and psychologically related symptoms of fear of falling and functional seizures than to other motor symptoms, such as weakness or balance impairment. This finding was not unexpected, given that pathophysiological models of FND, supported by imaging studies, describe abnormal connectivity between emotional processing areas of the brain and the primary motor cortex (Perez et al. 2021, Aybek et al. 2015). The motor/nonmotor binary division is reinforced by the language we use to describe symptoms and the structure of health care (physical therapies vs. psychological therapies). However, the symptoms of FND defy this dualism and the clinical implication is that interdisciplinary treatment is necessary for people with FGD. Interdisciplinary care goes beyond parallel workings of a multidisciplinary team, with therapists working in an integrated fashion on shared goals to improve patient outcomes (Lidstone, MacGillivray, and Lang 2020). Physiotherapy for FND is encouraged to be “psychologically informed,” and perhaps psychological therapies should look toward incorporating physical strategies (Perez et al. 2021) Secondly, when assessing and planning treatment for FGD, the assessment should take a broad view of potential contributing symptoms, among other factors.

This study has a number of limitations. Participants were self‐selected based on screening questions, which may have led to the inclusion of people not meeting eligibility. Participants self‐reported their symptoms which may have been a source of error, if they did not understand the definition of each symptom. To limit this issue, we defined each symptom in the survey. Our sample may not be representative of the wider population of people with FGD, given there was a larger than normal female bias (90.4%, commonly closer to 70%; Lidstone et al. 2022) and the fact that we did not collect data on the proportion of different cultural and racial identities represented within the cohort. Another factor limiting the generalizability of our cohort was that we did not employ any other methods in our recruitment to reach individuals with limited digital connectivity. Our respondents also had relatively long symptom duration, which may suggest the cohort was skewed toward people with treatment‐resistant symptoms. We completed multiple analyses to inform which variables entered our multivariable models, which increased the risk of finding significance by chance alone. As this was an exploratory study, adding corrections for multiple comparisons would have limited the maximal set of possible associations between variables and risk omitting symptoms of impact that need further exploration. As discussed earlier, we applied a distinction between motor and nonmotor symptoms, which may be a false distinction. Finally, our target population was people with FND and gait disorder, which may differ from those who are judged by a clinician as having FGD, with differences reported in the literature regarding the precise definition of FGD, such as a “pure” functional gait disorder (altered gait only) versus a mixed presentation (Nonnekes et al. 2020).

## 5 Conclusions

Motor and nonmotor symptoms were prevalent and severe among respondents with FGD. Motor and nonmotor symptoms were associated with dependent ambulation, reduced participation in work and social functions, and reduced physical and mental quality of life. This study provides researchers and clinicians with real‐world data about a large range of motor and nonmotor symptoms in people with FGD, which may inform assessment, patient education, and treatment. Additionally, it highlights the multidimensional nature of FGD and supports the need for interdisciplinary care. Future research may consider designing interventions that target these symptoms and examine treatment outcomes in people with FGD.

## Author Contributions

**Sara Issak**: conceptualization, investigation, funding acquisition, writing–original draft, methodology, validation, visualization, writing–review and editing, project administration, data curation, resources, formal analysis. **Gavin Williams**: conceptualization, methodology, writing–review and editing, supervision, visualization. **Richard A. Kanaan**: conceptualization, writing–review and editing, supervision, methodology, visualization. **Natalie A. Fini**: conceptualization, writing–review and editing, methodology, supervision, visualization. **Glenn Nielsen**: conceptualization, writing–review and editing, methodology, validation, supervision, visualization.

## Conflicts of Interest

The authors declare no conflicts of interest.

### Peer Review

The peer review history for this article is available at https://publons.com/publon/10.1002/brb3.70208

## Supporting information



Supporting Information

Supporting Information

Supporting Information

Table S1 ‐ Self‐reported comorbidities reported by survey respondents

Table S2 ‐ Re*sults from 36‐Item short form survey (SF36) questionnaire*

Table S3 ‐ *Results from the functional ambulation category (FAC)*

Table S4 ‐ *Results from the functional mobility scale (FMS)*

Table S5 ‐ *Associations between self‐reported symptoms and ambulation status*

Table S6 ‐ *Binary logistic regression analysis between constant symptoms and dependent ambulation*

Table S7 ‐ *Associations between self‐reported symptoms and participation in work and social functions*

Table S8 ‐ *Stepwise regression analysis of constant symptoms and participation in work and social functions*

Table S9 ‐ *Associations of self‐reported symptoms and physical quality of life*

Table S10 ‐ *Stepwise regression analysis of constant symptoms and physical‐QOL*

Table S11 ‐ *Associations of self‐reported symptoms and mental quality of life*

Table S12 ‐ *Stepwise regression analysis of constant symptoms and mental‐QOL*

Supporting Information

## Data Availability

The data that support the findings of this study are available in the Supporting Information of this article.
