# Supplementary material for: Self‐Reported Motor and Non‐Motor Symptoms in People With Functional Gait Disorder: A Cross‐Sectional Study
Source: Brain Behav. 2025 Feb 6;15(2):e70208. doi: 10.1002/brb3.70208 (PMC11802242; doi:10.1002/brb3.70208)
Supplement: Supplementary file 1 — Supporting Information [file BRB3-15-e70208-s013.pdf]

Survey questions

Thank you for completing those questions.

You can now commence the survey questions below

Section 2 - About your symptoms

This section of the survey will cover
1) Physical and non-physical symptoms that you are currently having
2) How severe those symptoms are
3) How frequently they occur
4) Which parts of the body are affected

The symptoms we are investigating include physical features, for example weakness or trembling of limbs, and non-physical features such as pain or fatigue

A few follow up questions may pop-up when/if you select certain features from the lists below

If you need a break during the survey, you can save and exit and return to it at a later time to complete it. Make sure to take note of the re-entry code that appears on your screen that you will require to re-enter the survey later.

Physical symptoms. Please tick from the following features those which apply to you and that you are currently experiencing.	☐ Weakness, or loss of strength, of the legs, arms or body<br>☐ Tremor in arms (trembling or shaking of the arms, legs or body)<br>☐ Jerks or sudden contraction of the arms, legs or body (myoclonus)<br>☐ Muscle spasms that cause abnormal movements or postures (dystonia)<br>☐ Stiffness causing difficulty moving (rigidity)<br>☐ Difficulty coordinating limb movements (including ataxia)<br>☐ Slowness of movement (bradykinesia)<br>☐ Reduced or altered sense of balance<br>☐ Other
Please describe the 'other' symptom you are currently experiencing	__________________________________
Can you describe where your current physical symptoms are?<br>Does it affect your body, legs or one side of your body? Please select the areas that apply to you	☐ right side of your body/torso<br>☐ left side of your body/torso<br>☐ right leg<br>☐ left leg<br>☐ right arm<br>☐ left arm<br>☐ head or neck

Weakness, or loss of strength, of the legs, arms or body

Please score this symptom from 0 (Not present) to 100 (Very severe)

Note, that if you are completing this on your tablet device, you may need to tap the line to see the options

not present weakness very severe

(Place a mark on the scale above)

Tremor in arms (trembling or shaking of the arms, legs or body)

Please score this symptom from 0 (Not present) to 100 (Very severe)

Note, that if you are completing this on your tablet device, you may need to tap the line to see the options

not present tremor very severe

(Place a mark on the scale above)

Jerks or sudden contraction of the arms, legs or body (myoclonus)

Please score this symptom from 0 (Not present) to 100 (Very severe)

Note, that if you are completing this on your tablet device, you may need to tap the line to see the options

not present jerks very severe

(Place a mark on the scale above)

Muscle spasms that cause abnormal movements or postures (dystonia)

Please score this symptom from 0 (Not present) to 100 (Very severe)

Note, that if you are completing this on your tablet device, you may need to tap the line to see the options

not present dystonia very severe

(Place a mark on the scale above)

Stiffness causing difficulty moving (rigidity)

Please score this symptom from 0 (Not present) to 100 (Very severe)

Note, that if you are completing this on your tablet device, you may need to tap the line to see the options

not present rigidity very severe

(Place a mark on the scale above)

Difficulty coordinating limb movements (including ataxia)

Please score this symptom from 0 (Not present) to 100 (Very severe)

Note, that if you are completing this on your tablet device, you may need to tap the line to see the options

not present coordination very severe

(Place a mark on the scale above)

Slowness of movement (bradykinesia)

Please score this symptom from 0 (Not present) to 100 (Very severe)

Note, that if you are completing this on your tablet device, you may need to tap the line to see the options

not present slowness very severe

(Place a mark on the scale above)

Reduced or altered sense of balance

Please score this symptom from 0 (Not present) to 100 (Very severe)

Note, that if you are completing this on your tablet device, you may need to tap the line to see the options

not present balance very severe

(Place a mark on the scale above)

Other symptom

Please score this symptom from 0 (Not present) to 100 (Very severe)

Note, that if you are completing this on your tablet device, you may need to tap the line to see the options

not present other symptom very severe

(Place a mark on the scale above)

Weakness of the legs, arms or body

How frequently does this feature occur/ how often do you experience it? Please pick the option that is closest.

◯ Constant (every hour of the day while I am awake) ◯ Most of the day
◯ Most days of the week
◯ Once a week ◯ Once a month
◯ Once every 3 months
◯ Only once or twice in the last 12 months
◯ Never

Tremor (trembling or shaking of the arms, legs or body)

How frequently does this feature occur/ how often do you experience it? Please pick the option that is closest.

◯ Constant (every hour of the day while I am awake) ◯ Most of the day
◯ Most days of the week
◯ Once a week ◯ Once a month
◯ Once every 3 months
◯ Only once or twice in the last 12 months
◯ Never

Jerks or sudden contraction of the arms, legs or body (myoclonus)<br><br>How frequently does this feature occur/ how often do you experience it? Please pick the option that is closest.	◯ Constant (every hour of the day while I am awake) ◯ Most of the day<br>◯ Most days of the week<br>◯ Once a week ◯ Once a month<br>◯ Once every 3 months<br>◯ Only once or twice in the last 12 months<br>◯ Never
Muscle spasms that cause abnormal movements or postures (dystonia)<br><br>How frequently does this feature occur/ how often do you experience it? Please pick the option that is closest.	◯ Constant (every hour of the day while I am awake) ◯ Most of the day<br>◯ Most days of the week<br>◯ Once a week ◯ Once a month<br>◯ Once every 3 months<br>◯ Only once or twice in the last 12 months<br>◯ Never
Stiffness causing difficulty moving (rigidity)<br><br>How frequently does this feature occur/ how often do you experience it? Please pick the option that is closest.	◯ Constant (every hour of the day while I am awake) ◯ Most of the day<br>◯ Most days of the week<br>◯ Once a week ◯ Once a month<br>◯ Once every 3 months<br>◯ Only once or twice in the last 12 months<br>◯ Never
Difficulty coordinating limb movements (including ataxia)<br><br>How frequently does this feature occur/ how often do you experience it? Please pick the option that is closest.	◯ Constant (every hour of the day while I am awake) ◯ Most of the day<br>◯ Most days of the week<br>◯ Once a week ◯ Once a month<br>◯ Once every 3 months<br>◯ Only once or twice in the last 12 months<br>◯ Never
Slowness of movement (bradykinesia)<br><br>How frequently does this feature occur/ how often do you experience it? Please pick the option that is closest.	◯ Constant (every hour of the day while I am awake) ◯ Most of the day<br>◯ Most days of the week<br>◯ Once a week ◯ Once a month<br>◯ Once every 3 months<br>◯ Only once or twice in the last 12 months<br>◯ Never
Reduced or altered sense of balance<br><br>How frequently does this feature occur/ how often do you experience it? Please pick the option that is closest.	◯ Constant (every hour of the day while I am awake) ◯ Most of the day<br>◯ Most days of the week<br>◯ Once a week ◯ Once a month<br>◯ Once every 3 months<br>◯ Only once or twice in the last 12 months<br>◯ Never
Other symptom<br><br>How frequently does this feature occur/ how often do you experience it? Please pick the option that is closest.	◯ Constant (every hour of the day while I am awake) ◯ Most of the day<br>◯ Most days of the week<br>◯ Once a week ◯ Once a month<br>◯ Once every 3 months<br>◯ Only once or twice in the last 12 months<br>◯ Never

The next section will cover a series of question about non-physical symptoms such as pain, fatigue and sensory changes

Non-physical symptoms: Please select from the following list of features those which apply to you and that you are currently experiencing.

- ☐ Pain
- ☐ Fatigue (for example, tiredness, exhaustion based on your usual level of fatigue)
- ☐ Fear of falling
- ☐ Fear of moving (Kinesiophobia)
- ☐ Anxiety
- ☐ Depression/Low mood
- ☐ Sensory symptoms (for example, loss or altered sensation in limbs or body, numbness or pins and needles)
- ☐ Headache or migraine
- ☐ Bowel/bladder dysfunction (for example, going a lot to the toilet, having to rush to get there, occasional accidents)
- ☐ Memory and cognitive trouble (for example, forgetting things, trouble multi-tasking or difficulty paying attention)
- ☐ Trouble with speech
- ☐ Trouble with swallow
- ☐ Seizures and/or blackouts (body shakes, collapsing)
- ☐ Dissociative symptoms (for example, a feeling that your body doesn't quite belong to you or is disconnected from you or that you are disconnected from the world around you or "spaced out")
- ☐ Dizziness or vertigo (for example, feeling as though the room is spinning around you or you are falling)
- ☐ Other
- ☐ Visual symptoms (changes to your vision)

Please describe the 'other' symptom you are currently experiencing

____

If you currently have pain, can you describe where your pain is?
Does it affect your body, legs or one side of your body? Please select the areas that apply to you

- ☐ right side of your body/torso
- ☐ left side of your body/torso
- ☐ right leg
- ☐ left leg
- ☐ right arm
- ☐ left arm
- ☐ head, neck or face

If you currently have sensory changes, can you describe where your altered sensation is?
Does it affect your body, legs or one side of your body? Please select the areas that apply to you

- ☐ right side of your body/torso
- ☐ left side of your body/torso
- ☐ right leg
- ☐ left leg
- ☐ right arm
- ☐ left arm
- ☐ head, neck or face

Pain

Please score this symptom from 0 (Not present) to 100 (Very severe)

Note, that if you are completing this on your tablet device, you may need to tap the line to see the options

not present — pain — very severe

(Place a mark on the scale above)

Fatigue (tiredness, exhaustion)

Please score this symptom from 0 (Not present) to 100 (Very severe)

Note, that if you are completing this on your tablet device, you may need to tap the line to see the options

not present | fatigue | very severe

(Place a mark on the scale above)

Fear of falling

Please score this symptom from 0 (Not present) to 100 (Very severe)

Note, that if you are completing this on your tablet device, you may need to tap the line to see the options

not present | fear of falling | very severe

(Place a mark on the scale above)

Fear of moving (Kinesiophobia)

Please score this symptom from 0 (Not present) to 100 (Very severe)

Note, that if you are completing this on your tablet device, you may need to tap the line to see the options

not present | fear of moving | very severe

(Place a mark on the scale above)

Anxiety

Please score this symptom from 0 (Not present) to 100 (Very severe)

Note, that if you are completing this on your tablet device, you may need to tap the line to see the options

not present | anxiety | very severe

(Place a mark on the scale above)

Sensory symptoms (for example loss of sensation, altered sensation in limbs or body, numbness or pins and needles)

Please score this symptom from 0 (Not present) to 100 (Very severe)

Note, that if you are completing this on your tablet device, you may need to tap the line to see the options

not present | sensory symptoms | very severe

(Place a mark on the scale above)

Headache or migraine

Please score this symptom from 0 (Not present) to 100 (Very severe)

Note, that if you are completing this on your tablet device, you may need to tap the line to see the options

not present | headache | very severe

(Place a mark on the scale above)

Bowel/bladder dysfunction

Please score this symptom from 0 (Not present) to 100 (Very severe)

Note, that if you are completing this on your tablet device, you may need to tap the line to see the options

not present | bowel and bladder | very severe

(Place a mark on the scale above)

Memory and cognitive trouble

Please score this symptom from 0 (Not present) to 100 (Very severe)

Note, that if you are completing this on your tablet device, you may need to tap the line to see the options

not present | cognitive symptoms | very severe

(Place a mark on the scale above)

Trouble with speech

Please score this symptom from 0 (Not present) to 100 (Very severe)

Note, that if you are completing this on your tablet device, you may need to tap the line to see the options

not present | speech trouble | very severe

(Place a mark on the scale above)

Trouble with swallowing

Please score this symptom from 0 (Not present) to 100 (Very severe)

Note, that if you are completing this on your tablet device, you may need to tap the line to see the options

not present | swallowing issues | very severe

(Place a mark on the scale above)

Seizures and/or blackouts

Please score this symptom from 0 (Not present) to 100 (Very severe)

Note, that if you are completing this on your tablet device, you may need to tap the line to see the options

not present seizures very severe

(Place a mark on the scale above)

Dissociative symptoms (these can include a feeling that your body doesn't quite belong to you or is disconnected from you or that you are disconnected from the world around you or "spaced out")

Please score this symptom from 0 (Not present) to 100 (Very severe)

Note, that if you are completing this on your tablet device, you may need to tap the line to see the options

not present dissociation very severe

(Place a mark on the scale above)

Dizziness or vertigo (feeling as though the room is spinning around you or you are falling)

Please score this symptom from 0 (Not present) to 100 (Very severe)

Note, that if you are completing this on your tablet device, you may need to tap the line to see the options

not present dizziness very severe

(Place a mark on the scale above)

Other non-physical symptom

Please score this symptom from 0 (Not present) to 100 (Very severe)

Note, that if you are completing this on your tablet device, you may need to tap the line to see the options

not present other non-physical symptom very severe

(Place a mark on the scale above)

Visual changes

Please score this symptoms from 0 (Not present) to 100 (Very severe)

Note that if you are completing this on your tablet device you may need to tap the line to see the options

not present visual changes very severe

(Place a mark on the scale above)

Pain

How frequently does this feature occur/ how often do you experience it? Please pick the option that is closest.

◯ Constant (every hour of the day while I am awake)
◯ Most of the day
◯ Most days of the week
◯ Once a week
◯ Once a month
◯ Once every 3 months
◯ Only once or twice in the last 12 months
◯ Never

Fatigue (tiredness, exhaustion)

How frequently does this feature occur/ how often do you experience it? Please pick the option that is closest.

◯ Constant (every hour of the day while I am awake)
◯ Most of the day
◯ Most days of the week
◯ Once a week
◯ Once a month
◯ Once every 3 months
◯ Only once or twice in the last 12 months
◯ Never

Fear of falling

How frequently does this feature occur/ how often do you experience it? Please pick the option that is closest.

◯ Constant (every hour of the day while I am awake)
◯ Most of the day
◯ Most days of the week
◯ Once a week
◯ Once a month
◯ Once every 3 months
◯ Only once or twice in the last 12 months
◯ Never

Fear of moving (Kinesiophobia)

How frequently does this feature occur/ how often do you experience it? Please pick the option that is closest.

◯ Constant (every hour of the day while I am awake)
◯ Most of the day
◯ Most days of the week
◯ Once a week
◯ Once a month
◯ Once every 3 months
◯ Only once or twice in the last 12 months
◯ Never

Anxiety

How frequently does this feature occur/ how often do you experience it? Please pick the option that is closest.

◯ Constant (every hour of the day while I am awake)
◯ Most of the day
◯ Most days of the week
◯ Once a week
◯ Once a month
◯ Once every 3 months
◯ Only once or twice in the last 12 months
◯ Never

Depression/Low mood

How frequently does this feature occur/ how often do you experience it? Please pick the option that is closest.

◯ Constant (every hour of the day while I am awake)
◯ Most of the day
◯ Most days of the week
◯ Once a week
◯ Once a month
◯ Once every 3 months
◯ Only once or twice in the last 12 months
◯ Never

Sensory symptoms (for example loss or altered sensation in limbs or body, numbness or pins and needles)<br><br>How frequently does this feature occur/ how often do you experience it? Please pick the option that is closest.	◯ Constant (every hour of the day while I am awake)<br>◯ Most of the day<br>◯ Most days of the week<br>◯ Once a week<br>◯ Once a month<br>◯ Once every 3 months<br>◯ Only once or twice in the last 12 months<br>◯ Never
Headache or migraine<br><br>How frequently does this feature occur/ how often do you experience it? Please pick the option that is closest.	◯ Constant (every hour of the day while I am awake)<br>◯ Most of the day<br>◯ Most days of the week<br>◯ Once a week<br>◯ Once a month<br>◯ Once every 3 months<br>◯ Only once or twice in the last 12 months<br>◯ Never
Bowel/bladder dysfunction (going a lot to the toilet, having to rush to get there, occasional accidents)<br><br>How frequently does this feature occur/ how often do you experience it? Please pick the option that is closest.	◯ Constant (every hour of the day while I am awake)<br>◯ Most of the day<br>◯ Most days of the week<br>◯ Once a week<br>◯ Once a month<br>◯ Once every 3 months<br>◯ Only once or twice in the last 12 months<br>◯ Never
Memory and cognitive trouble (for example, forgetting things, trouble with problem solving or multi-tasking)<br><br>How frequently does this feature occur/ how often do you experience it? Please pick the option that is closest.	◯ Constant (every hour of the day while I am awake)<br>◯ Most of the day<br>◯ Most days of the week<br>◯ Once a week<br>◯ Once a month<br>◯ Once every 3 months<br>◯ Only once or twice in the last 12 months<br>◯ Never
Trouble with speech<br><br>How frequently does this feature occur/ how often do you experience it? Please pick the option that is closest.	◯ Constant (every hour of the day while I am awake)<br>◯ Most of the day<br>◯ Most days of the week<br>◯ Once a week<br>◯ Once a month<br>◯ Once every 3 months<br>◯ Only once or twice in the last 12 months<br>◯ Never
Trouble with swallowing<br><br>How frequently does this feature occur/ how often do you experience it? Please pick the option that is closest.	◯ Constant (every hour of the day while I am awake)<br>◯ Most of the day<br>◯ Most days of the week<br>◯ Once a week<br>◯ Once a month<br>◯ Once every 3 months<br>◯ Only once or twice in the last 12 months<br>◯ Never

Seizures and/or blackouts (body shakes, collapsing)<br><br>How frequently does this feature occur/ how often do you experience it? Please pick the option that is closest.	◯ Constant (every hour of the day while I am awake)<br>◯ Most of the day<br>◯ Most days of the week<br>◯ Once a week<br>◯ Once a month<br>◯ Once every 3 months<br>◯ Only once or twice in the last 12 months<br>◯ Never
Dissociative symptoms (these can include a feeling that your body doesn't quite belong to you or is disconnected from you or that you are disconnected from the world around you or "spaced out")<br><br>How frequently does this feature occur/ how often do you experience it? Please pick the option that is closest.	◯ Constant (every hour of the day while I am awake)<br>◯ Most of the day<br>◯ Most days of the week<br>◯ Once a week<br>◯ Once a month<br>◯ Once every 3 months<br>◯ Only once or twice in the last 12 months<br>◯ Never
Dizziness or vertigo (feeling as though the room is spinning around you or you are falling)<br><br>How frequently does this feature occur/ how often do you experience it? Please pick the option that is closest.	◯ Constant (every hour of the day while I am awake)<br>◯ Most of the day<br>◯ Most days of the week<br>◯ Once a week<br>◯ Once a month<br>◯ Once every 3 months<br>◯ Only once or twice in the last 12 months<br>◯ Never
Other non-physical symptom<br><br>How frequently does this feature occur/ how often do you experience it? Please pick the option that is closest.	◯ Constant (every hour of the day while I am awake)<br>◯ Most of the day<br>◯ Most days of the week<br>◯ Once a week<br>◯ Once a month<br>◯ Once every 3 months<br>◯ Only once or twice in the last 12 months<br>◯ Never
Visual changes<br><br>How frequently does this feature occur/how often do you experience it?<br>Please pick the closest option.	◯ Constant (every hour of the day while I am awake)<br>◯ Most of the day<br>◯ Most days of the week<br>◯ Once a week<br>◯ Once a month<br>◯ Once every 3 months<br>◯ Only once or twice in the last 12 months<br>◯ Never

The next part of the survey will include a series of questionnaires to further explore particular symptoms. We are going to ask you about these symptoms even if you didn't choose them earlier in the survey.

Now we would like to ask some questions about how concerned you are about the possibility of falling. For each of the following activities, please circle the opinion closest to your own to show how concerned you are that you might fall if you did this activity. Please reply thinking about how you usually do the activity. If you currently don't do the activity (e.g. if someone does your shopping for you), please answer to show whether you think you would be concerned about falling IF you did the activity.

	Not at all concerned	Somewhat concerned	Fairly concerned	Very concerned
Cleaning the house (eg: sweep, dust, vacuum)	○	○	○	○
Getting dressed	○	○	○	○
Preparing simple meals	○	○	○	○
Taking a bath or shower	○	○	○	○
Going to the shop	○	○	○	○
Getting in or out of a chair	○	○	○	○
Going up or down stairs	○	○	○	○
Walking around the neighborhood	○	○	○	○
Reaching for something above your head or on the ground	○	○	○	○
Going to answer the phone before it stops ringing	○	○	○	○
Walking on a slippery surface (e.g. wet or icy)	○	○	○	○
Visiting a friend or relative	○	○	○	○
Walking in a place with crowds	○	○	○	○
Walking on an uneven surface (Rocky ground, poorly maintained pavement)	○	○	○	○
Walking up or down a slope	○	○	○	○
Going out to a social event (family gathering, religious service, club meeting)	○	○	○	○

FSES Total

____

Hospital anxiety and depression scale
Please answer the following questions about how you have been feeling this past week. Often your immediate response is best, try not to over think the answers

I feel tense or 'wound up'	◯ Most of the time<br>◯ A lot of the time<br>◯ From time to time, occasionally<br>◯ Not at all
I still enjoy the things I used to enjoy	◯ Definitely as much<br>◯ Not quite so much<br>◯ Only a little<br>◯ Not at all
I get a sort of frightened feeling like something awful is about to happen	◯ Very definitely and quite badly<br>◯ Yes, but not too badly<br>◯ A little, but it doesn't worry me<br>◯ Not at all
I can laugh and see the funny side of things	◯ As much as I always could<br>◯ Not quite so much now<br>◯ Definitely not so much now<br>◯ Not at all
Worrying thoughts go through my mind	◯ A great deal of the time<br>◯ A lot of the time<br>◯ From time to time but not too often<br>◯ Only occasionally
I feel cheerful	◯ Not at all<br>◯ Not often<br>◯ Sometimes<br>◯ Most of the time
I can sit at ease and feel relaxed	◯ Definitely<br>◯ Usually<br>◯ Not often<br>◯ Not at all
I feel as if I am slowed down	◯ Nearly all of the time<br>◯ Very often<br>◯ Sometimes<br>◯ Not at all
I get a sort of frightened feeling like 'butterflies in the stomach'	◯ Not at all<br>◯ Occasionally<br>◯ Quite often<br>◯ Very often
I have lost interest in my appearance	◯ Definitely<br>◯ I don't take as much care as I should<br>◯ I may not take quite as much care<br>◯ I take just as much care as ever
I feel restless as if I have to be on the move	◯ Very much indeed<br>◯ Quite a lot<br>◯ Not very much<br>◯ Not at all

I look forward with enjoyment to things	◯ As much as I ever did<br>◯ Rather less than I used to<br>◯ Definitely less than I used to<br>◯ Hardly at all
I get sudden feelings of panic	◯ Very often indeed<br>◯ Quite often<br>◯ Not very often<br>◯ Not at all
I can enjoy a good book or radio or TV programme	◯ Often<br>◯ Sometimes<br>◯ Not often<br>◯ Very seldom
Anxiety score	______________________<br>(from HADS)
Depression score	______________________<br>(from HADS)
HADS Total	______________________

Tampa scale for fear of moving
This questionnaire will explore the fear of movement or the fear of injury from moving

	strongly disagree	disagree	agree	strongly agree
I'm afraid I might injure myself if I exercise	○	○	○	○
If I were to try and overcome it, my pain would increase	○	○	○	○
My body is telling me I have something dangerously wrong	○	○	○	○
My pain would probably be relieved if I exercise	○	○	○	○
People aren't taking my medical condition seriously enough	○	○	○	○
My injury has put my body at risk for the rest of my life	○	○	○	○
Pain always means I have injured my body	○	○	○	○
Just because something aggravates my pain does not mean it is dangerous	○	○	○	○
I am afraid that I might injure myself accidentally	○	○	○	○
Simply being careful that I do not make any unnecessary movements is the safest thing I can do to prevent my pain from worsening	○	○	○	○
I wouldn't have this much pain if there weren't something potentially dangerous going on in my body	○	○	○	○
Although my condition is painful, I would be better off if I were physically active	○	○	○	○
Pain lets me know when to stop exercising so that I don't injure myself	○	○	○	○
It's really not safe for a person with a condition like mine to be physically active	○	○	○	○
I can't do all the things normal people do because it's too easy for me to get injured	○	○	○	○

Even though something is causing me a lot of pain, I don't think it's actually dangerous	○	○	○	○
No one should have to exercise when he/she is in pain	○	○	○	○

Tampa Score ______________________________

Physical symptoms questionnaire

During the past 4 weeks, how much have you been bothered by any of the following problems?

	Not bothered at all	Bothered a little	Bothered a lot
a. Stomach pain	○	○	○
b. Back pain	○	○	○
c. Pain in your arms, legs, or joints (knees, hips, etc.)	○	○	○
d. Menstrual cramps or other problems with your periods (WOMEN ONLY)	○	○	○
e. Headaches	○	○	○
f. Chest pain	○	○	○
g. Dizziness	○	○	○
h. Fainting spells	○	○	○
i. Feeling your heart pound or race	○	○	○
j. Shortness of breath	○	○	○
k. Pain or problems during sexual intercourse	○	○	○
l. Constipation, loose bowels, or diarrhea	○	○	○
m. Nausea, gas, or indigestion	○	○	○
n. Feeling tired or having low energy	○	○	○
o. Trouble sleeping	○	○	○

Score ________________________________

Section 3: The impact of your functional gait disorder on your walking and everyday life

The next section will explore the impact of your functional gait disorder on your walking, daily activities, and quality of life. Please complete the following questions

Functional ambulation category
Please select the option below that best describes your current walking ability.

- ◯ 0 Non-functional ambulation = Need help from more than one person to walk OR cannot walk
- ◯ 1 Dependent Level 2 = Need help from one person to walk all the time
- ◯ 2 Dependent Level 1 = Need help from one person some of the time
- ◯ 3 Dependent with supervision = Need supervision from another person to walk but no physical help
- ◯ 4 Independent level surfaces = Can walk on flat surfaces. May need supervision on stairs, hills, or uneven surfaces
- ◯ 5 Independent = Can walk on flat and uneven surfaces

Functional mobility scale

If you had to walk 5 metres (5.4 yards)...

Select the number from 1-6 that describes your current function for this distance. If you cannot walk the distance, then select N = does not apply

- ◯ 1 = uses a wheelchair, or may stand for transfers, or do some stepping supported by another person, or use a walking frame
- ◯ 2 = uses a walking frame without help from another person
- ◯ 3 = uses crutches without help from another person
- ◯ 4 = uses walking sticks (one or two) without the help from another person or uses furniture/walls for support
- ◯ 5 = independent on all surfaces without any walking aids, needs the rails going up/down stairs
- ◯ 6 = independent on all surfaces without any walking aids, including outdoors and crowded areas
- ◯ N = does not apply

Functional mobility scale

If you had to walk 50 metres (54 yards)...

Select the number from 1-6 that describes your current function for this distance. If you cannot walk the distance, then select N = does not apply

- ◯ 1 = uses a wheelchair, or may stand for transfers, or do some stepping supported by another person, or use a walking frame
- ◯ 2 = uses a walking frame without help from another person
- ◯ 3 = uses crutches without help from another person
- ◯ 4 = uses walking sticks (one or two) without the help from another person or uses furniture/walls for support
- ◯ 5 = independent on all surfaces without any walking aids, needs the rails going up/down stairs
- ◯ 6 = independent on all surfaces without any walking aids, including outdoors and crowded areas
- ◯ N = does not apply

Functional mobility scale

If you had to walk 500 metres (546 yards)...

Select the number from 1-6 that describes your current function for this distance. If you cannot walk the distance, then select N = does not apply

- ◯ 1 = uses a wheelchair, or may stand for transfers, or do some stepping supported by another person, or use a walking frame
- ◯ 2 = uses a walking frame without help from another person
- ◯ 3 = uses crutches without help from another person
- ◯ 4 = uses walking sticks (one or two) without the help from another person or uses furniture/walls for support
- ◯ 5 = independent on all surfaces without any walking aids, needs the rails going up/down stairs
- ◯ 6 = independent on all surfaces without any walking aids, including outdoors and crowded areas
- ◯ N = does not apply

36-Item Short Form Survey Instrument

This questionnaire will ask questions related to your quality of life

Choose one option for each questionnaire item

In general, would you say your health is:	◯ Excellent<br>◯ Very good<br>◯ Good<br>◯ Fair<br>◯ Poor
Score	__________________________________
Compared to one year ago, how would you rate your health in general now?	◯ Much better now than one year ago<br>◯ Somewhat better now than one year ago<br>◯ About the same<br>◯ Somewhat worse now than one year ago<br>◯ Much worse now than one year ago
Score	__________________________________

The following questions are about activities you might do during a typical day. Does your health now limit you in these activities. If so, how much?

Vigorous activities, such as running, lifting heavy objects, participating in strenuous sports	◯ Yes, limited a lot<br>◯ Yes, limited a little<br>◯ Not limited at all
Score	__________________________________
Moderate activities such as moving a table, pushing a vacuum cleaner, bowling or playing	◯ Yes, limited a lot<br>◯ Yes, limited a little<br>◯ Not limited at all
Score	__________________________________
Lifting or carrying groceries	◯ Yes, limited a lot<br>◯ Yes, limited a little<br>◯ Not limited at all
Score	__________________________________
Climbing SEVERAL flights of stairs	◯ Yes, limited a lot<br>◯ Yes, limited a little<br>◯ Not limited at all
Score	__________________________________

Climbing ONE flight of stairs	◯ Yes, limited a lot<br>◯ Yes, limited a little<br>◯ Not limited at all
Score	______________________
Bending, kneeling or stooping	◯ Yes, limited a lot<br>◯ Yes, limited a little<br>◯ Not limited at all
Score	______________________
Walking more than a mile	◯ Yes, limited a lot<br>◯ Yes, limited a little<br>◯ Not limited at all
Score	______________________
Walking several blocks	◯ Yes, limited a lot<br>◯ Yes, limited a little<br>◯ Not limited at all
Score	______________________
Walking one block	◯ Yes, limited a lot<br>◯ Yes, limited a little<br>◯ Not limited at all
Score	______________________
Bathing or dressing yourself	◯ Yes, limited a lot<br>◯ Yes, limited a little<br>◯ Not limited at all
Score	______________________

During the past 4 weeks, how much of the time have you had any of the following problems with your work or other daily activities as a result of your physical health

Cut down on the amount of time you spent on work or other activities	◯ Yes<br>◯ No
Score	______________________
Accomplished less than you would like	◯ Yes<br>◯ No
Score	______________________

Were limited in the kind of work or other activities	◯ Yes<br>◯ No
Score	__________________________________
Had difficulty performing the work or other activities (e.g. it took extra effort)	◯ Yes<br>◯ No
Score	__________________________________
During the past 4 weeks, how much of the time have you had any of the following problems with your work other other regular daily activities as a result of any emotional problems (such as anxiety or depression)?	
Cut down on the amount of time you spent on work or other activities	◯ Yes<br>◯ No
Score	__________________________________
Accomplished less than you would have liked	◯ Yes<br>◯ No
Score	__________________________________
Didn't do work or other activities as carefully as usual	◯ Yes<br>◯ No
Score	__________________________________
During the past 4 weeks, to what extent has your physical health or emotional problems interfered with your normal social activities with family, friends, neighbours or groups?	◯ Not at all<br>◯ Slightly<br>◯ Moderately<br>◯ Quite a bit<br>◯ Extremely
Score	__________________________________
How much bodily pain have you had during the past 4 weeks	◯ None<br>◯ Very mild<br>◯ Mild<br>◯ Moderate<br>◯ Severe<br>◯ Very Severe
Score	__________________________________

During the past 4 weeks, how much did pain interfere with your normal work (including both work outside the home and housework)	◯ Not at all<br>◯ A little bit<br>◯ Moderately<br>◯ Quite a bit<br>◯ Extremely
Score	______________________

These questions are about how you feel and how things have been with you during the past 4 weeks. For each question please give the one answer that comes closest to the way you have been feeling.
How much of the time in the past 4 weeks...

Did you feel full of life?	◯ All of the time<br>◯ Most of the time<br>◯ A good bit of the time<br>◯ Some of the time<br>◯ A little of the time<br>◯ None of the time
Score	______________________
Have you been very nervous?	◯ All of the time<br>◯ Most of the time<br>◯ A good bit of the time<br>◯ Some of the time<br>◯ A little of the time<br>◯ None of the time
Score	______________________
Have you felt so down in the dumps that nothing could cheer you up?	◯ All of the time<br>◯ Most of the time<br>◯ A good bit of the time<br>◯ Some of the time<br>◯ A little of the time<br>◯ None of the time
Score	______________________
Have you felt calm and peaceful?	◯ All of the time<br>◯ Most of the time<br>◯ A good bit of the time<br>◯ Some of the time<br>◯ A little of the time<br>◯ None of the time
Score	______________________

Did you have a lot of energy?	◯ All of the time<br>◯ Most of the time<br>◯ A good bit of the time<br>◯ Some of the time<br>◯ A little of the time<br>◯ None of the time
Score	__________________________________
Have you felt downhearted and depressed?	◯ All of the time<br>◯ Most of the time<br>◯ A good bit of the time<br>◯ Some of the time<br>◯ A little of the time<br>◯ None of the time
Score	__________________________________
Did you feel worn out?	◯ All of the time<br>◯ Most of the time<br>◯ A good bit of the time<br>◯ Some of the time<br>◯ A little of the time<br>◯ None of the time
Score	__________________________________
Have you been happy?	◯ All of the time<br>◯ Most of the time<br>◯ A good bit of the time<br>◯ Some of the time<br>◯ A little of the time<br>◯ None of the time
Score	__________________________________
Did you feel tired?	◯ All of the time<br>◯ Most of the time<br>◯ A good bit of the time<br>◯ Some of the time<br>◯ A little of the time<br>◯ None of the time
Score	__________________________________
During the past 4 weeks, how much of the time has your physical health or emotional problems interfere with your social activities (like visiting friends, relatives etc)?	◯ All of the time<br>◯ Most of the time<br>◯ Some of the time<br>◯ A little of the time<br>◯ None of the time
Score	__________________________________

How TRUE or FALSE is each of the following statements for you

I seem to get sick a little easier than other people	◯ Definitely true<br>◯ Mostly true<br>◯ Don't know<br>◯ Mostly false<br>◯ Definitely false
Score	______________________
I am as healthy as anybody I know	◯ Definitely true<br>◯ Mostly true<br>◯ Don't know<br>◯ Mostly false<br>◯ Definitely false
Score	______________________
I expect my health to get worse	◯ Definitely true<br>◯ Mostly true<br>◯ Don't know<br>◯ Mostly false<br>◯ Definitely false
Score	______________________
My health is excellent	◯ Definitely true<br>◯ Mostly true<br>◯ Don't know<br>◯ Mostly false<br>◯ Definitely false
Score	______________________
SF36 Score (Physical Functioning component)	______________________
SF36 Score (Role Limitations due to Physical Health component)	______________________
SF36 Score (Role Limitations due to Emotional Problems component)	______________________
SF36 Score (Energy/Fatigue component)	______________________
SF36 Score (Emotional Well-being component)	______________________
SF36 Score (Social Functioning component)	______________________

SF36 Score (Pain component)

____

SF36 Score (General Health component)

____

Work and social scale

People's problems sometimes affect their ability to do certain day to day tasks. Look at the following questions and determine how much your problem impairs your ability to carry out the activity

Because of my symptoms, my ability to work is impaired.	◯ 0 (Not at all) ◯ 1 ◯ 2 (Slightly) ◯ 3 ◯ 4 (Definitely) ◯ 5 ◯ 6 (Markedly) ◯ 7 ◯ 8 (Very severely - can't work)
Because of my symptoms, my home management (cleaning, tidying, shopping, cooking, looking after home/children, paying bills) is impaired.	◯ 0 (Not at all) ◯ 1 ◯ 2 (Slightly) ◯ 3 ◯ 4 (Definitely) ◯ 5 ◯ 6 (Markedly) ◯ 7 ◯ 8 (Very severely)
Because of my symptoms, my social leisure activities (with others e.g. parties, bars, clubs, outings, visits, dating, home entertainment) are is impaired.	◯ 0 (Not at all) ◯ 1 ◯ 2 (Slightly) ◯ 3 ◯ 4 (Definitely) ◯ 5 ◯ 6 (Markedly) ◯ 7 ◯ 8 (Very severely)
Because of my symptoms, my private leisure activities (done alone e.g. reading, gardening, sewing, walking alone) are is impaired.	◯ 0 (Not at all) ◯ 1 ◯ 2 (Slightly) ◯ 3 ◯ 4 (Definitely) ◯ 5 ◯ 6 (Markedly) ◯ 7 ◯ 8 (Very severely)
Because of my symptoms, my close relationships with others, including those I live with, is impaired.	◯ 0 (Not at all) ◯ 1 ◯ 2 (Slightly) ◯ 3 ◯ 4 (Definitely) ◯ 5 ◯ 6 (Markedly) ◯ 7 ◯ 8 (Very severely)
WSAS Total	________________________ ((out of 40))
