# Supplementary material for: Self‐Reported Motor and Non‐Motor Symptoms in People With Functional Gait Disorder: A Cross‐Sectional Study
Source: Brain Behav. 2025 Feb 6;15(2):e70208. doi: 10.1002/brb3.70208 (PMC11802242; doi:10.1002/brb3.70208)
Supplement: Supplementary file 2 — Supporting Information [file BRB3-15-e70208-s003.pdf]

PLAIN LANGUAGE SUMMARY – PATIENT INFORMATION FORM

THE UNIVERSITY OF MELBOURNE

Observational Study - *Adult providing own consent*

**Title**	Self-reported symptoms of Functional Gait Disorders
**Project Number:**	2021-22778-23028-2
**Researchers:**	**Primary investigator:** Professor Gavin Williams, Epworth Healthcare and The University of Melbourne<br><br>**Associate Investigators:**<br><br>Sara Issak, Department of Physiotherapy, Epworth Healthcare, Department of Physiotherapy, School of Health Sciences, The University of Melbourne<br><br>Professor Richard Kanaan, Department of Psychiatry, Austin Health, The University of Melbourne<br><br>Dr Natalie Fini, Department of Physiotherapy, School of Health Sciences, The University of Melbourne<br><br>Dr Glenn Nielsen, Neurosciences Research Centre, Molecular & Clinical Sciences Research Institute, St George's, University of London
**Location**	Online recruitment. The University of Melbourne

1 Introduction

Functional Neurological Disorders (FND) is a common condition that causes neurological symptoms such as weakness, difficulty with walking or sensory loss, which is not due to structural damage in the brain, such as in stroke. A Functional Neurological Disorder that affects walking is called Functional Gait Disorder (FGD) and is a common problem in FND.

As you may have experienced, a variety of different symptoms may affect movement, such as weakness, muscle stiffness, trembling or trouble with balance. Non-motor symptoms such as pain, fatigue, or fear of falling are also common. It is not clear how often each of these symptoms occur, how severely they impact someone's ability to walk or their quality of life.

Please read the following information carefully. You can contact us to ask questions about anything that you don't understand or want to know more about. Before deciding whether or not to take part, you might want to talk about your participation in the study with a relative, friend or your preferred health care worker.

2 What is the purpose of this research?

We plan to explore the nature and extent of symptoms that occur in functional gait disorders to identify what the most common problems are and the impact on individuals' daily life.

3 Who can get involved in this research?

Adult participants who are aged 18 or over can get involved if they have received a formal diagnosis of FND and self-identify as having a functional gait disorder (altered walking). In addition, participants will need to be able to complete online surveys and questionnaires in English.

Participants who are unable to walk at all, with or without a walking aid or another person's assistance, and need bed-based care, will not be eligible to participate in this study.

4 What does participation in this research involve and what do I have to do?

This research has 3 parts. You may participate in 1, 2 or 3 parts of this project. Firstly, participation will involve the completion of an online survey that will take approximately 45 minutes. Secondly, people who complete the first survey will be asked to participate in follow up surveys 3-,6- and 12 months later. If you complete the first survey, it is not compulsory to complete the repeated surveys. Finally, you can upload a short video of your walking to be used by our research team to further understand functional gait disorders.

5 Do I have to take part in this research project?

Participation in this research is entirely voluntary. You are welcome to participate if you are interested in this research project. You will need to indicate that you have read this information and agree to participate in the survey before proceeding. You are free to withdraw at any time if you change your mind.

6 What are the possible benefits of taking part?

Participating in this research may benefit the community and people with functional gait disorders. There are no direct benefits of participating in this project, however, to compensate you for your time, an online MasterCard voucher valued at $30 AUD will be emailed to participants. You will have the option to opt out of this payment and have the funds donated to future research of functional gait disorders if you wish.

7 What are the possible risks and disadvantages of taking part?

We understand when participating in this research there is a chance that you may become upset while recalling your symptoms, or experience fatigue due to answering questions.
You can withdraw from the study at any point if you feel distressed. If you feel distressed as a result of the study, you are encouraged to contact:

- The Principal Investigator of this study
- Your General Practitioner

AND/OR

- Beyond Blue (available 24 hours a day) on Ph:1300 224 636
- Mental Health Line on Ph:1800 011 511

8 What if I withdraw from this research project?

If you decide to withdraw from the project, the researchers would like to keep the information about you that has already been collected. This is to ensure that the results of the research can be properly reported.

Your decision to withdraw will not affect your relationship with the researchers, the university, or any affiliated health provider in any way.

9 What happens when the research project ends?

We are planning to use the information from this study to publish written findings from the study to assist clinicians who treat people with FGD. The study findings will be presented at national & international research conferences.

10 What will happen to information about me?

By signing the consent form, you consent to the research team collecting and using personal information about you for the research project.

Any information obtained in connection with this research project that can identify you will remain confidential and be securely stored. Information will be kept in a password protected online database, which only the study investigators can access. Your information will only be used for the purpose of this research project, and it will only be disclosed with your permission, or to comply with the law.

It is anticipated that the results of this research project will be used to plan future research projects and may be presented in a variety of forums. In any publication and/or presentation, information will be provided in such a way that you cannot be identified.

In accordance with relevant Australian and/or State of Victoria privacy laws, you have the right to request access to your information collected and stored by the research team.

This information will be stored for 5 years. After this time the information will be destroyed and access to your specific information will not be possible.

11 Further information and who to contact

If you want any further information concerning this project, you can contact:

Professor Gavin Williams
gavinw@unimelb.edu.au
+61 3 9426 8727

or

Sara Issak
sara.issak@student.unimelb.edu.au
+61 3 9805 4178

12 Complaints

This research project has been approved by the Human Research Ethics Committee of The University of Melbourne. If you have any concerns or complaints about the conduct of this research project, which you do not wish to discuss with the research team, you should contact the Manager, Human Research Ethics, Office for Research Ethics and Integrity, University of Melbourne, VIC 3010. Tel: +61 3 8344 2073 or email: research-integrity@unimelb.edu.au

All complaints will be treated confidentially. In any correspondence, please provide the name of the research team or the ethics ID number of the research project.
