# Supplementary material for: Self‐Reported Motor and Non‐Motor Symptoms in People With Functional Gait Disorder: A Cross‐Sectional Study
Source: Brain Behav. 2025 Feb 6;15(2):e70208. doi: 10.1002/brb3.70208 (PMC11802242; doi:10.1002/brb3.70208)
Supplement: Supplementary file 3 — Supporting Information [file BRB3-15-e70208-s014.docx]

**Legend of supplementary information**

| **Supporting information** | **Details and format** |
| --- | --- |
| File_S1_SuppInfo.pdf | Copy of plain language summary – patient information form, PDF file |
| File_S2_SuppInfor.pdf | Copy of survey questions, PDF file |
| Table_S1_SuppInfo | Self-reported comorbidities reported by survey respondents, word file |
| Table_S2_SuppInfo | Results from 36-Item short form survey (SF36) questionnaire, word file |
| Table_S3_SuppInfo | Results from the functional ambulation category (FAC), word file |
| Table_S4_SuppInfo | Results from the functional mobility scale (FMS), word file |
| Table_S5_SuppInfo | Associations between self-reported symptoms and ambulation status, word file |
| Table_S6_SuppInfo | Binary logistic regression analysis between constant symptoms and dependent ambulation, word file |
| Table_S7_SuppInfo | Associations between self-reported symptoms and participation in work and social functions, word file |
| Table_S8_SuppInfo | Stepwise regression analysis of constant symptoms and participation in work and social functions, word file |
| Table_S9_SuppInfo | Associations of self-reported symptoms and physical quality of life, word file |
| Table_S10_SuppInfo | Stepwise regression analysis of constant symptoms and physical-QOL, word file |
| Table_S11_SuppInfo | Associations of self-reported symptoms and mental quality of life, word file |
| Table_S12_SuppInfo | Stepwise regression analysis of constant symptoms and mental-QOL, word file |
| Table_S13_SuppInfo | Symptom prevalence correlation matrix |
