# Supplementary material for: Self‐Reported Motor and Non‐Motor Symptoms in People With Functional Gait Disorder: A Cross‐Sectional Study
Source: Brain Behav. 2025 Feb 6;15(2):e70208. doi: 10.1002/brb3.70208 (PMC11802242; doi:10.1002/brb3.70208)
Supplement: Supplementary file 4 — Table S1 ‐ Self‐reported comorbidities reported by survey respondents [file BRB3-15-e70208-s012.docx]

**Table S1 - *Self-reported comorbidities reported by survey respondents***

| **Comorbidity** | ***n*** | ***%*** |
| --- | --- | --- |
| **Sciatica** | 9 | 5.8 |
| **Ehlers-Danlos Syndrome** | 4 | 2.6 |
| **Osteoarthritis** | 3 | 1.9 |
| **Chronic back pain** | 2 | 1.3 |
| **Fibromyalgia** | 2 | 1.3 |
| **Parkinson's Disease** | 1 | 0.6 |
| **Multiple Sclerosis** | 1 | 0.6 |
| **Rheumatoid Arthritis** | 1 | 0.6 |
| **Ankylosing Spondylitis** | 1 | 0.6 |
| **Developmental co-ordination disorder** | 1 | 0.6 |
| **Pes planus** | 1 | 0.6 |
| **Familial Mediterranean fever** | 1 | 0.6 |
| **Lumbar spondylosis** | 1 | 0.6 |
| **Multi nodule goitre** | 1 | 0.6 |
| **Myasthenia gravis** | 1 | 0.6 |
| **Generalised dystonia** | 1 | 0.6 |
| **Hemiplegic migraines** | 1 | 0.6 |
| **Peripheral Neuropathy** | 1 | 0.6 |
| **Hip joint effusion** | 1 | 0.6 |
| **Psoriatic arthritis** | 1 | 0.6 |
| **Knee injury** | 1 | 0.6 |
| **Lumbar radiculopathy** | 1 | 0.6 |
| **Scleroderma** | 1 | 0.6 |
| **Myalgic encephalomyelitis** | 1 | 0.6 |
| **Meniere’s disease** | 1 | 0.6 |
