# Supplementary material for: Self‐Reported Motor and Non‐Motor Symptoms in People With Functional Gait Disorder: A Cross‐Sectional Study
Source: Brain Behav. 2025 Feb 6;15(2):e70208. doi: 10.1002/brb3.70208 (PMC11802242; doi:10.1002/brb3.70208)
Supplement: Supplementary file 5 — Table S2 ‐ Results from 36‐Item short form survey (SF36) questionnaire [file BRB3-15-e70208-s011.docx]

**Table S2 - Re*sults from 36-Item short form survey (SF36) questionnaire***

| **Domain** | ***n*** | ***%*** | ***mean*** | **±*SD*** |
| --- | --- | --- | --- | --- |
| **Physical function** | 128 | 82.1 | 30.9 | 24.8 |
| **Limitations due to physical function** | 127 | 81.4 | 8.5 | 21.2 |
| **Emotional problems** | 127 | 81.4 | 30.9 | 38.5 |
| **Energy** | 127 | 81.4 | 21.4 | 19.1 |
| **Wellbeing** | 127 | 81.4 | 50.5 | 23.9 |
| **Social function** | 127 | 81.4 | 32.3 | 27.6 |
| **Pain** | 127 | 81.4 | 38.5 | 27.9 |
| **General health perceptions** | 128 | 82.1 | 33.7 | 21.8 |
