# Supplementary material for: Self‐Reported Motor and Non‐Motor Symptoms in People With Functional Gait Disorder: A Cross‐Sectional Study
Source: Brain Behav. 2025 Feb 6;15(2):e70208. doi: 10.1002/brb3.70208 (PMC11802242; doi:10.1002/brb3.70208)
Supplement: Supplementary file 6 — Table S3 ‐ Results from the functional ambulation category (FAC) [file BRB3-15-e70208-s006.docx]

**Table S3 - *Results from the functional ambulation category (FAC)***

| **Score** | ***n*** | ***%*** |
| --- | --- | --- |
| **0; Need help from 2 people to walk (Non-functional ambulation)** | 4 | 3.1 |
| **1; Need help from one person all time (Dependent level 2)** | 12 | 9.4 |
| **2; Need help from one person some of the time (Dependent level 1)** | 29 | 22.7 |
| **3; Need supervision from another to walk (Dependent with supervision)** | 15 | 11.7 |
| **4; Can walk on flat surfaces, needs help on stairs/uneven ground (Independent level surfaces)** | 37 | 28.9 |
| **5; Can walk on flat and uneven surfaces (Independent all surfaces)** | 31 | 24.2 |
