# Supplementary material for: Self‐Reported Motor and Non‐Motor Symptoms in People With Functional Gait Disorder: A Cross‐Sectional Study
Source: Brain Behav. 2025 Feb 6;15(2):e70208. doi: 10.1002/brb3.70208 (PMC11802242; doi:10.1002/brb3.70208)
Supplement: Supplementary file 7 — Table S4 ‐ Results from the functional mobility scale (FMS) [file BRB3-15-e70208-s016.docx]

**Table S4 - *Results from the functional mobility scale (FMS)***

| **Score** | **Distance** | | | | | |
| --- | --- | --- | --- | --- | --- | --- |
|  | **5 metres** | | **50 metres** | | **500 metres** | |
|  | ***n*** | ***%*** | ***n*** | ***%*** | ***n*** | ***%*** |
| **1; Uses a wheelchair, stepping supported by another person or uses a walking frame** | 15 | 11.7 | 19 | 14.8 | 37 | 28.9 |
| **2; Uses a walking frame without help from another person** | 12 | 9.4 | 16 | 12.5 | 9 | 7.0 |
| **3; Uses crutches without help from another person** | 9 | 7 | 7 | 5.5 | 6 | 4.7 |
| **4; Uses walking sticks (one or two) without help from another person or uses furniture/walls** | 28 | 21.9 | 28 | 21.9 | 23 | 18.0 |
| **5; Indep on all surfaces without walking aids but needs rails on stairs** | 41 | 32.0 | 29 | 22.7 | 21 | 16.4 |
| **6; Indep on all surfaces without walking aids** | 20 | 15.6 | 17 | 13.3 | 14 | 10.9 |
| **N; Does not apply/cannot walk this distance** | 3 | 2.3 | 12 | 9.4 | 18 | 14.1 |
