# Supplementary material for: Self‐Reported Motor and Non‐Motor Symptoms in People With Functional Gait Disorder: A Cross‐Sectional Study
Source: Brain Behav. 2025 Feb 6;15(2):e70208. doi: 10.1002/brb3.70208 (PMC11802242; doi:10.1002/brb3.70208)
Supplement: Supplementary file 8 — Table S5 ‐ Associations between self‐reported symptoms and ambulation status [file BRB3-15-e70208-s002.docx]

**Table S5 - *Associations between self-reported symptoms and ambulation status***

| **Symptom** | **Dependent**  **Ambulation, n (%)** | **Independent Ambulation, n (%)** | ***X^2^ (df)*** | ***p value*** | | | ***phi (effect size)*** |
| --- | --- | --- | --- | --- | --- | --- | --- |
| **Motor symptoms** |  |  |  | |  |  |  |
| **Constant weakness** | 53 (88.3) | 45 (66.2) | 7.529 (1) | | .006 | .261 | |
| **Episodic weakness** | 7 (11.7) | 23 (33.8) |  | |  |  | |
| **Constant reduced balance** | 47 (78.3) | 38 (55.9) | 6.231 (1) | | .013 | .237 | |
| **Episodic reduced balance** | 13 (21.7) | 30 (44.1) |  | |  |  | |
| **Constant tremor** | 39 (65.0) | 23 (33.8) | 11.188 (1) | | <.001 | .311 | |
| **Episodic tremor** | 21 (35.0) | 45 (66.2) |  | |  |  | |
| **Constant jerks** | 38 (63.3) | 27 (39.7) | 6.206 (1) | | .013 | .236 | |
| **Episodic jerks** | 22 (36.7) | 41 (60.3) |  | |  |  | |
| **Constant ataxia** | 36 (60.0) | 28 (41.2) | 3.796 | | .051 | .188 | |
| **Episodic ataxia** | 24 (40.0) | 40 (58.8) |  | |  |  | |
| **Constant dystonia** | 34 (56.7) | 21 (30.9) | 7.627 | | .006 | .260 | |
| **Episodic dystonia** | 26 (43.3) | 47 (69.1) |  | |  |  | |
| **Constant bradykinesia** | 37 (61.7) | 23 (33.8) | 8.836 | | .003 | .278 | |
| **Episodic bradykinesia** | 23 (38.3) | 45 (66.2) |  | |  |  | |
| **Constant rigidity** | 25 (41.7) | 24 (35.3) | .311 | | .577 | .065 | |
| **Episodic rigidity** | 35 (58.3) | 44 (64.7) |  | |  |  | |
| **Non-motor symptoms** |  |  |  | |  |  | |
| **Constant fatigue** | 58 (96.7) | 57 (83.8) | 4.440 | | .035 | .212 | |
| **Episodic fatigue** | 2 (3.3) | 11 (16.2) |  | |  |  | |
| **Constant somatosensory** | 40 (66.7) | 44 (64.7) | .002 | | .963 | .021 | |
| **Episodic somatosensory** | 20 (33.3) | 24 (35.3) |  | |  |  | |
| **Constant cognitive** | 46 (76.7) | 41 (60.3) | 3.209 | | .073 | .175 | |
| **Episodic cognitive** | 14 (23.3) | 27 (39.7) |  | |  |  | |
| **Constant pain** | 46 (76.7) | 40 (58.8) | 3.829 | | .051 | .190 | |
| **Episodic pain** | 14 (23.3) | 28 (41.2) |  | |  |  | |
| **Constant anxiety** | 41 (68.3) | 32 (47.1) | 5.051 | | .025 | .214 | |
| **Episodic anxiety** | 19 (31.7) | 36 (52.9) |  | |  |  | |
| **Constant depression** | 31 (51.7) | 24 (35.3) | 2.851 | | .091 | .165 | |
| **Episodic depression** | 29 (48.3) | 44 (64.7) |  | |  |  | |
| **Contant speech** | 27 (45.0) | 22 (32.4) | 1.656 | | .198 | .130 | |
| **Episodic speech** | 33 (55.0) | 46 (67.6) |  | |  |  | |
| **Constant dissociation** | 26 (43.3) | 20 (29.4) | 2.113 | | .146 | .145 | |
| **Episodic dissociation** | 34 (56.7) | 48 (70.6) |  | |  |  | |
| **Constant dizziness** | 27 (45.0) | 19 (27.9) | 3.322 | | .068 | .177 | |
| **Episodic dizziness** | 33 (55.0) | 49 (72.1) |  | |  |  | |
| **Constant headache** | 19 (31.7) | 19 (27.9) | .071 | | .790 | .041 | |
| **Episodic headache** | 41 (68.3) | 49 (72.1) |  | |  |  | |
| **Constant visual** | 20 (33.3) | 23 (33.8) | .000 | | 1.00 | -.005 | |
| **Episodic visual** | 40 (66.7) | 45 (66.2) |  | |  |  | |
| **Constant fear of falling** | 30 (50.0) | 12 (17.6) | 13.702 | | <.001 | .344 | |
| **Episodic fear of falling** | 30 (50.0) | 56 (82.4) |  | |  |  | |
| **Constant bowel/bladder** | 24 (40.0) | 20 (29.4) | 1.150 | | .284 | .111 | |
| **Episodic bowel/bladder** | 36 (60.0) | 48 (70.6) |  | |  |  | |
| **Constant seizures** | 17 (28.3) | 2 (2.9) | 14.312 | | <.001 | .356 | |
| **Episodic seizures** | 43 (71.7) | 66 (97.1) |  | |  |  | |
| **Constant swallowing** | 14 (23.3) | 8 (11.8) | 2.239 | | .135 | .153 | |
| **Episodic swallowing** | 46 (76.7) | 60 (88.2) |  | |  |  | |
| **Constant kinesiophobia** | 6 (10.0) | 1 (1.5) | 2.987 | | .084 | .187 | |
| **Episodic kinesiophobia** | 54 (90.0) | 67 (98.5) |  | |  |  | |

**Note. Chi square test for independence was conducted with data from 128 respondents’ functional ambulation category results. A 2x2 cross-tabulation was conducted with constant or episodic symptoms, and dependent or independent ambulation as measured by the FAC. Chi-square test for independence, with Yates continuity correction value, was used as each variable only had 2 categories.**
