# Supplementary material for: Self‐Reported Motor and Non‐Motor Symptoms in People With Functional Gait Disorder: A Cross‐Sectional Study
Source: Brain Behav. 2025 Feb 6;15(2):e70208. doi: 10.1002/brb3.70208 (PMC11802242; doi:10.1002/brb3.70208)
Supplement: Supplementary file 9 — Table S6 ‐ Binary logistic regression analysis between constant symptoms and dependent ambulation [file BRB3-15-e70208-s004.docx]

**Table S6 - *Binary logistic regression analysis between constant symptoms and dependent ambulation***

| **Step 1^a^** | **B** | ***SE*** | **p** | **OR** | **95% CI** | |
| --- | --- | --- | --- | --- | --- | --- |
| **Variable** |  |  |  |  |  |  |
| **Weakness** | -.485 | .595 | .415 | .616 | .192 | 1.976 |
| **Tremor** | -.500 | .472 | .289 | .607 | .241 | 1.529 |
| **Jerks** | -.087 | .467 | .852 | .916 | .367 | 2.287 |
| **Dystonia** | -.406 | .498 | .415 | .666 | .251 | 1.769 |
| **Ataxia** | .123 | .464 | .790 | 1.131 | .456 | 2.806 |
| **Bradykinesia** | -.453 | .455 | .319 | .636 | .261 | 1.549 |
| **Balance** | -.182 | .505 | .719 | .834 | .310 | 2.244 |
| **Fatigue** | -.728 | .864 | .399 | .483 | .089 | 2.624 |
| **Fear of falling** | -1.019 | .490 | .038 | .361 | .138 | .943 |
| **Anxiety** | -.457 | .444 | .303 | .633 | .265 | 1.510 |
| **Functional seizures** | -1.819 | .844 | .031 | .162 | .031 | .848 |

**^a.^ Variables entered on step 1: Constant weakness, tremor, jerks, dystonia, ataxia, bradykinesia, balance impairment, fatigue, fear of falling, anxiety and functional seizures. B = estimated logit coefficient, SE = standard error of the coefficient, OR = Odds ratio, CI = Confidence interval.**
