# Supplementary material for: Self‐Reported Motor and Non‐Motor Symptoms in People With Functional Gait Disorder: A Cross‐Sectional Study
Source: Brain Behav. 2025 Feb 6;15(2):e70208. doi: 10.1002/brb3.70208 (PMC11802242; doi:10.1002/brb3.70208)
Supplement: Supplementary file 10 — Table S7 ‐ Associations between self‐reported symptoms and participation in work and social functions [file BRB3-15-e70208-s015.docx]

**Table S7 - *Associations between self-reported symptoms and participation in work and social functions***

| **Symptom** | ***Mean WSAS score*** | | | | ***t*** | | **p** | | **95% CI** | | **Cohens D** | |  |
| --- | --- | --- | --- | --- | --- | --- | --- | --- | --- | --- | --- | --- | --- |
|  | **Constant symptoms** | | **Episodic symptoms** | |  | |  | |  | |  | |  |
| **Motor symptoms** |  | |  | |  | |  | |  | |  | |  |
| **Weakness** | 27.24 | | 19.28 | | 3.984 | | <.001 | | [4.01, 11.93] | | .842 | |  |
| **Rigidity** | 30.22 | | 22.41 | | 4.617 | | <.001 | | [4.47, 11.16] | | .842 | |  |
| **Tremor** | 29.18 | | 21.85 | | 4.421 | | <.001 | | [4.05, 10.61] | | .785 | |  |
| **Jerks** | 28.80 | | 21.89 | | 4.133 | | <.001 | | [3.60, 10.22] | | .734 | |  |
| **Bradykinesia** | 29.30 | | 21.96 | | 4.424 | | <.001 | | [4.06, 10.63] | | .786 | |  |
| **Reduced balance** | 27.44 | | 21.49 | | 3.294 | | =.001 | | [2.38, 9.53] | | .618 | |  |
| **Dystonia** | 28.69 | | 22.93 | | 3.342 | | =.001 | | [2.35, 9.17] | | .599 | |  |
| **Ataxia** | 28.27 | | 22.63 | | 3.301 | | =.001 | | [2.26, 9.02] | | .586 | |  |
| **Other motor** | 30.33 | | 24.91 | | 1.802 | | .074 | | [-.53, 11.37] | | .547 | |  |
| Non-motor symptoms | |  | |  | |  | |  | |  | |  | |
| **Functional seizures** | 34.84 | | 23.77 | | 4.826 | | <.001 | | [6.53, 15.61] | | 1.201 | |  |
| **Kinesiophobia** | 35.57 | | 24.83 | | 2.836 | | .005 | | [3.25, 18.23] | | 1.103 | |  |
| **Pain** | 28.12 | | 19.78 | | 4.751 | | <.001 | | [4.86, 11.80] | | .902 | |  |
| **Fatigue** | 26.22 | | 17.83 | | 2.839 | | .005 | | [2.53, 14.23] | | .861 | |  |
| **Speech** | 30.14 | | 22.46 | | 4.526 | | <.001 | | [4.32, 11.04] | | .825 | |  |
| **Fear of falling** | 30.19 | | 23.07 | | 3.990 | | <.001 | | [3.59, 10.65] | | .753 | |  |
| **Bowel and/or bladder** | 30.00 | | 23.00 | | 3.965 | | <.001 | | [3.50, 10.49] | | .739 | |  |
| **Depression** | 28.98 | | 22.71 | | 3.671 | | <.001 | | [2.89, 9.67] | | .657 | |  |
| **Anxiety** | 27.79 | | 22.22 | | 3.216 | | .002 | | [2.14, 9.00] | | .577 | |  |
| **Cognitive** | 27.23 | | 21.63 | | 3.043 | | .003 | | [1.96, 9.24] | | .578 | |  |
| **Visual** | 28.93 | | 23.63 | | 2.907 | | .004 | | [1.69, 8.91] | | .545 | |  |
| **Swallowing** | 29.50 | | 24.57 | | 2.130 | | .035 | | [.35, 9.51] | | .499 | |  |
| **Dizziness** | 28.50 | | 23.68 | | 2.673 | | .009 | | [1.25, 8.39] | | .493 | |  |
| **Dissociation** | 28.26 | | 23.81 | | 2.454 | | .015 | | [.86, 8.03] | | .453 | |  |
| **Headache** | 27.82 | | 24.40 | | 1.774 | | .078 | | [-.39, 7.22] | | .344 | |  |
| **Somatosensory** | 26.51 | | 23.30 | | 1.724 | | .087 | | [-.48, 6.89] | | .323 | |  |
| **Other non-motor** | 28.00 | | 25.16 | | .936 | | .351 | | [-3.17, 8.85] | | .284 | |  |

**Note. An independent samples t-test was conducted on a total sample of 127 respondents who completed the work and social adjustment scale (WSAS). A comparison of WSAS mean differences (response variable) between constant vs episodic symptom groups was conducted (grouping variable).**
