# Supplementary material for: Self‐Reported Motor and Non‐Motor Symptoms in People With Functional Gait Disorder: A Cross‐Sectional Study
Source: Brain Behav. 2025 Feb 6;15(2):e70208. doi: 10.1002/brb3.70208 (PMC11802242; doi:10.1002/brb3.70208)
Supplement: Supplementary file 11 — Table S8 ‐ Stepwise regression analysis of constant symptoms and participation in work and social functions [file BRB3-15-e70208-s010.docx]

**Table S8 - *Stepwise regression analysis of constant symptoms and participation in work and social functions***

| **Symptom/predictor** | **Model 1** | | **Model 2** | | **Model 3** | | **Model 4** | | **Model 5** | | **Model 6** | |
| --- | --- | --- | --- | --- | --- | --- | --- | --- | --- | --- | --- | --- |
|  | **β** | ***p*** | **β** | ***p*** | **β** | ***p*** | **β** | **p** | **β** | **p** | **β** | ***p*** |
| **Functional seizures** | -.396 | <.001 | -.342 | <.001 | -.307 | <.001 | -.294 | <.001 | -.262 | <.001 | -.231 | .002 |
| **Rigidity** |  |  | -.325 | <.001 | -.333 | <.001 | -.298 | <.001 | -.239 | .002 | -.191 | .017 |
| **Depression** |  |  |  |  | -.276 | <.001 | -.235 | .002 | -.209 | .006 | -.189 | .012 |
| **Fear of falling** |  |  |  |  |  |  | -.185 | .016 | -.183 | .015 | -.174 | .019 |
| **Pain** |  |  |  |  |  |  |  |  | -.190 | .016 | -.191 | .014 |
| **Speech symptoms** |  |  |  |  |  |  |  |  |  |  | -.162 | .037 |
| **Adjusted R^2^** | .150 | | .248 | | .319 | | .345 | | .371 | | .388 | |
| **F for change in R^2^** | 23.295 | | 21.741 | | 20.636 | | 17.590 | | 15.836 | | 14.314 | |

**Note. Dependent Variable was the total WSAS score. β = standardised coefficient beta**
