# Supplementary material for: Self‐Reported Motor and Non‐Motor Symptoms in People With Functional Gait Disorder: A Cross‐Sectional Study
Source: Brain Behav. 2025 Feb 6;15(2):e70208. doi: 10.1002/brb3.70208 (PMC11802242; doi:10.1002/brb3.70208)
Supplement: Supplementary file 12 — Table S9 ‐ Associations of self‐reported symptoms and physical quality of life [file BRB3-15-e70208-s007.docx]

**Table S9 - *Associations of self-reported symptoms and physical quality of life***

| **Symptom** | ***Physical-QOL mean score*** | | ***t*** | **p** | **95% CI** | **Cohens D** |
| --- | --- | --- | --- | --- | --- | --- |
|  | **Constant symptom**  **group** | **Episodic symptom group** |  |  |  |  |
| **Motor symptoms** |  |  |  |  |  |  |
| **Bradykinesia** | 25.95 | 32.19 | -4.570 | <.001 | [-8.93,-3.53] | -.812 |
| **Rigidity** | 25.98 | 31.29 | -3.703 | <.001 | [-8.15,-2.47] | -.675 |
| **Weakness** | 28.09 | 22.12 | -2.971 | .004 | [-8.38, -1.68] | -.628 |
| **Jerks** | 26.86 | 31.73 | -3.465 | <.001 | [-7.65, -2.08] | -.615 |
| **Reduced balance** | 27.71 | 32.23 | -3.010 | =.003 | [-7.48,-1.54] | -.564 |
| **Dystonia** | 26.80 | 31.10 | -2.999 | .003 | [-7.15,-1.46] | -.537 |
| **Tremor** | 27.09 | 31.29 | -2.959 | .004 | [-7.0, -1.39] | -.525 |
| **Ataxia** | 27.36 | 31.09 | -2.600 | .010 | [-6.56, -.89] | -.461 |
| **Other motor** | 26.06 | 29.57 | -1.407 | .162 | [-8.44,1.43] | -.427 |
| **Non-motor symptoms** |  |  |  |  |  |  |
| **Fatigue** | 28.29 | 38.47 | -4.348 | <.001 | [-14.83,-5.55] | -1.319 |
| **Pain** | 26.74 | 34.49 | -5.487 | <.001 | [-10.54, -4.95] | -1.041 |
| **Functional seizures** | 23.37 | 30.27 | -3.512 | <.001 | [-10.80, -3.02] | -.874 |
| **Bowel and/or bladder symptoms** | 25.88 | 31.02 | -3.482 | <.001 | [-8.06,-2.22] | -.649 |
| **Visual symptoms** | 26.44 | 30.68 | -2.811 | .006 | [-7.21, -1.25] | -.527 |
| **Fear of falling** | 26.51 | 30.59 | -2.681 | .008 | [-7.08,-1.06] | -.506 |
| **Headache** | 26.57 | 30.38 | -2.427 | .017 | [-6.91, -.70] | -.470 |
| **Dizziness** | 26.91 | 30.56 | -2.443 | .016 | [-6.61, -.69] | -.451 |
| **Swallowing symptoms** | 25.98 | 29.92 | -2.061 | .041 | [-7.72, .15] | -.483 |
| **Speech symptoms** | 27.06 | 30.61 | -2.405 | .018 | [-6.48, -.63] | -.438 |
| **Fear of moving (kinesiophobia)** | 25.47 | 29.46 | -1.246 | .215 | [-10.33, 2.35] | -.485 |
| **Somatosensory Symptoms (e.g., paraesthesia)** | 28.21 | 31.24 | -1.980 | .050 | [-6.06, -.002] | -.371 |
| **Other non-motor symptoms** | 26.78 | 29.49 | -1.008 | .279 | [-7.67, 2.23] | -.330 |
| **Cognitive symptoms (e.g., memory issues)** | 28.35 | 31.09 | -1.767 | .080 | [-5.82, .33] | -.335 |
| **Depression** | 29.43 | 29.09 | .223 | .824 | [-2.61, 3.26] | -.040 |
| **Anxiety** | 30.08 | 28.10 | 1.344 | .182 | [-.94, 4.91] | .241 |
| **Dissociation** | 29.12 | 29.31 | -.126 | .900 | [-3.22, 2.83] | -.023 |

**Note.** **An independent samples t-test was conducted on a total sample of 127 respondents who completed the SF36 questionnaire. Mean physical-QOL summary scores (response variable) where compared between each motor and non-motor symptom (constant/episodic = grouping variable).**
