# Supplementary material for: Self‐Reported Motor and Non‐Motor Symptoms in People With Functional Gait Disorder: A Cross‐Sectional Study
Source: Brain Behav. 2025 Feb 6;15(2):e70208. doi: 10.1002/brb3.70208 (PMC11802242; doi:10.1002/brb3.70208)
Supplement: Supplementary file 13 — Table S10 ‐ Stepwise regression analysis of constant symptoms and physical‐QOL [file BRB3-15-e70208-s005.docx]

**Table S10 - *Stepwise regression analysis of constant symptoms and physical-QOL***

| **Symptom/predictor** | **Model 1** | | **Model 2** | | **Model 3** | | **Model 4** | |
| --- | --- | --- | --- | --- | --- | --- | --- | --- |
|  | **β** | ***p*** | **β** | ***p*** | **β** | ***p*** | **β** | **p** |
| **Pain** | .441 | <.001 | .377 | <.001 | .320 | <.001 | .292 | <.001 |
| **Bradykinesia** |  |  | .297 | <.001 | .267 | <.001 | .253 | .001 |
| **Fatigue** |  |  |  |  | .215 | .007 | .216 | .006 |
| **Dystonia** |  |  |  |  |  |  | .156 | .041 |
| **Adjusted R^2^** | .188 | | .267 | | .303 | | .321 | |
| **F for change in R^2^** | 30.111 | | 23.934 | | 19.288 | | 15.924 | |

**Note. Dependent Variable was the physical-QOL summary score (SF36). β = standardised coefficient beta**
