# Supplementary material for: Self‐Reported Motor and Non‐Motor Symptoms in People With Functional Gait Disorder: A Cross‐Sectional Study
Source: Brain Behav. 2025 Feb 6;15(2):e70208. doi: 10.1002/brb3.70208 (PMC11802242; doi:10.1002/brb3.70208)
Supplement: Supplementary file 14 — Table S11 ‐ Associations of self‐reported symptoms and mental quality of life [file BRB3-15-e70208-s009.docx]

**Table S11 - *Associations of self-reported symptoms and mental quality of life***

| **Symptom** | ***Mental-QOL mean score*** | | ***t*** | **p** | **95% CI** | **Cohens D** |
| --- | --- | --- | --- | --- | --- | --- |
|  | **Constant symptom group** | **Episodic symptom group** |  |  |  |  |
| **Motor symptoms** |  |  |  |  |  |  |
| **Jerks** | 31.63 | 36.21 | -2.077 | .040 | [-8.95,-.22] | -.369 |
| **Other motor** | 28.22 | 34.45 | -1.643 | .103 | [-13.74, 1.27] | -.498 |
| **Ataxia** | 31.86 | 35.84 | -1.796 | .075 | [-8.36, .40] | -.319 |
| **Weakness** | 33.04 | 36.64 | -1.356 | .178 | [-8.85,1.65] | -.287 |
| **Tremor** | 32.26 | 35.39 | -1.409 | .161 | [-7.55,1.27] | -.250 |
| **Bradykinesia** | 32.56 | 35.03 | -1.108 | .270 | [-6.90, 1.95] | -.197 |
| **Reduced balance** | 33.07 | 35.41 | -.993 | .323 | [-7.02, 2.33] | -.186 |
| **Rigidity** | 33.48 | 34.11 | -.273 | .786 | [-5.19,3.93] | -.050 |
| **Dystonia** | 33.79 | 33.91 | -.052 | .959 | [-4.59,4.36] | -.009 |
| **Non-motor symptoms** |  |  |  |  |  |  |
| **Depression** | 25.53 | 40.23 | -7.966 | <.001 | [-18.34, -11.04] | -1.428 |
| **Fear of moving (kinesiophobia)** | 18.92 | 34.74 | -3.359 | .001 | [-25.14, -6.50] | -1.306 |
| **Anxiety** | 28.07 | 41.69 | -7.118 | <.001 | [-17.42, -9.84] | -1.28 |
| **Functional seizures** | 26.03 | 35.24 | -3.034 | .003 | [-15.22, -3.20] | -.755 |
| **Fear of falling** | 29.69 | 35.93 | -2.692 | .008 | [-10.83,-1.65] | -.508 |
| **Dissociation** | 29.97 | 36.07 | -2.688 | .008 | [-10.59, -1.60] | -.496 |
| **Cognitive** | 31.95 | 37.89 | -2.538 | .012 | [-10.57, -1.31] | -.482 |
| **Speech** | 30.92 | 35.71 | -2.115 | .036 | [-9.27, -.31] | -.386 |
| **Fatigue** | 33.46 | 37.70 | -1.111 | .269 | [-11.79, 3.32] | -.337 |
| **Pain** | 32.69 | 36.32 | -1.523 | .130 | [-8.33, 1.08] | -.289 |
| **Bowel and/or bladder** | 32.09 | 34.80 | -1.155 | .250 | [-7.35, 1.93] | -.215 |
| **Other non-motor** | 31.66 | 34.09 | -.636 | .526 | [-10.01, 5.15] | -.193 |
| **Somatosensory** | 34.40 | 32.82 | .670 | .504 | [-3.09, 6.27] | .126 |
| **Dizziness** | 33.47 | 34.09 | -.264 | .792 | [-5.24, 4.00] | -.049 |
| **Headache** | 33.55 | 33.99 | -.180 | .857 | [-5.29, 4.41] | -.035 |
| **Visual symptoms** | 33.77 | 33.91 | -.057 | .955 | [-4.82, 4.56] | -.011 |
| **Swallowing symptoms** | 33.98 | 33.84 | .049 | .961 | [-5.72, 6.01] | .011 |

**Note.** **An independent samples t-test was conducted on a total sample of 127 respondents who completed the SF36 questionnaire. Mean mental-QOL summary scores (response variable) where compared between each motor and non-motor symptom (constant/episodic = grouping variable).**
