# Supplementary material for: Self‐Reported Motor and Non‐Motor Symptoms in People With Functional Gait Disorder: A Cross‐Sectional Study
Source: Brain Behav. 2025 Feb 6;15(2):e70208. doi: 10.1002/brb3.70208 (PMC11802242; doi:10.1002/brb3.70208)
Supplement: Supplementary file 15 — Table S12 ‐ Stepwise regression analysis of constant symptoms and mental‐QOL [file BRB3-15-e70208-s008.docx]

**Table S12 - *Stepwise regression analysis of constant symptoms and mental-QOL***

| **Symptom/predictor** | **Model 1** | | **Model 2** | | **Model 3** | |
| --- | --- | --- | --- | --- | --- | --- |
|  | **β** | ***p*** | **β** | ***p*** | **β** | ***p*** |
| **Depression** | .580 | <.001 | .436 | <.001 | .422 | <.001 |
| **Anxiety** |  |  | .364 | <.001 | .347 | <.001 |
| **Functional Seizures** |  |  |  |  | .162 | .016 |
| **Adjusted R^2^** | .331 | | .439 | | .461 | |
| **F for change in R^2^** | 63.465 | | 50.326 | | 36.898 | |

**Note. Dependent Variable was the mental-QOL summary score (SF36). β = standardised coefficient beta**
