# Supplementary material for: Self‐Reported Motor and Non‐Motor Symptoms in People With Functional Gait Disorder: A Cross‐Sectional Study
Source: Brain Behav. 2025 Feb 6;15(2):e70208. doi: 10.1002/brb3.70208 (PMC11802242; doi:10.1002/brb3.70208)
Supplement: Supplementary file 16 — Supporting Information [file BRB3-15-e70208-s001.pdf]

	weakness	tremor	myoclonus	dystonia	rigidity	ataxia	bradykinesia	reduced balance	pain	fatigue	fear of falling	fear of moving	anxiety	depression	sensory	headache	bowel and bladder	cognitive	speech	swallowing	seizures	dissociation	dizziness	visual
weakness	1																						
tremor	0.172	1																					
myoclonus	0.123	0.489	1																				
dystonia	0.084	0.384	0.543	1																			
rigidity	0.242	0.225	0.278	0.24	1																		
ataxia	0.036	0.198	0.308	0.254	0.226	1																	
bradykinesia	0.264	0.214	0.268	0.131	0.408	0.296	1																
reduced balance	0.306	0.201	0.154	0.171	0.17	0.205	0.229	1															
pain	0.273	0.26	0.293	0.195	0.306	0.109	0.238	0.130	1														
fatigue	0.206	0.096	0.123	0.084	0.205	0.036	0.19	0.121	0.465	1													
fear of falling	0.123	0.064	0.112	0.031	0.164	0.108	0.199	0.199	0.181	0.31	1												
kinesiophobia	0.100	0.083	0.032	0.110	0.125	0.039	0.113	0.054	0.071	0.021	0.233	1											
anxiety	0.186	0.144	0.228	0.123	0.147	0.232	0.086	0.238	0.191	0.336	0.292	0.150	1										
depression	0.163	0.083	0.084	0.021	0.037	0.006	0.061	0.100	0.243	0.347	0.279	0.183	0.449	1									
sensory	0.216	0.055	0.123	0.147	0.272	0.049	0.222	0.198	0.382	0.456	0.251	0.103	0.219	0.179	1								
headache	0.211	0.164	0.241	0.248	0.181	0.057	0.132	0.26	0.462	0.321	0.178	0.034	0.318	0.194	0.277	1							
bowel and bladder	0.226	0.204	0.145	0.139	0.296	0.139	0.386	0.252	0.434	0.301	0.077	0.073	0.113	0.177	0.377	0.204	1						
cognitive	0.336	0.17	0.266	0.118	0.16	0.191	0.278	0.268	0.353	0.497	0.28	0.043	0.418	0.375	0.3	0.333	0.292	1					
speech	0.195	0.31	0.283	0.242	0.307	0.178	0.307	0.061	0.26	0.342	0.108	0.021	0.308	0.166	0.282	0.309	0.239	0.366	1				
swallowing	0.096	0.131	0.114	0.254	0.288	0.251	0.109	0.051	0.135	0.183	0.163	0.121	0.159	0.024	0.169	0.090	0.148	0.103	0.393	1			
seizures	0.106	0.289	0.305	0.219	0.204	0.207	0.283	0.187	0.317	0.266	0.229	0.137	0.207	0.156	0.157	0.143	0.248	0.279	0.333	0.159	1		
dissociation	0.184	0.237	0.183	0.168	0.205	0.130	0.232	0.209	0.294	0.368	0.130	-0.027	0.235	0.218	0.377	0.308	0.313	0.405	0.282	0.166	0.349	1	
dizziness	0.29	0.069	0.199	0.105	0.193	0.120	0.272	0.267	0.391	0.363	0.271	0.086	0.224	0.206	0.201	0.397	0.245	0.341	0.218	0.263	0.33	0.295	1	
visual	0.208	0.137	0.291	0.236	0.291	0.181	0.247	0.243	0.307	0.319	0.268	0.060	0.234	0.199	0.267	0.359	0.239	0.324	0.417	0.27	0.183	0.233	0.399	1
